# Fullerene-decorated PdCo nano-resistor network hydrogen sensors with sub-second response and parts-per-billion detection at room temperature

**DOI:** 10.1038/s41467-025-67708-2

**Published:** 2025-12-19

**Authors:** Tu Anh Ngo, Ashwin T. Magar, Minh T. Pham, Hoang M. Luong, Thi Thu Trinh Phan, M. Tuan Trinh, Michael Jung, George K. Larsen, Yiping Zhao, Tho D. Nguyen

**Affiliations:** 1 Department of Physics and Astronomy, University of Georgia, Athens, GA USA; 2 Department of Electrical Engineering, Faculty of Engineering, Chulalongkorn University, Bangkok, Thailand; 3 Department of Chemistry and Biochemistry, Utah State University, Logan, UT USA; 4 School of Environmental, Civil, Agricultural and Mechanical Engineering, University of Georgia, Athens, GA USA; 5 Hydrogen Isotope Science Group, Savannah River National Laboratory, Aiken, SC USA

**Keywords:** Carbon nanotubes and fullerenes, Hydrogen storage materials, Nanosensors

## Abstract

Hydrogen detection with rapid response and ultra-low detection limits remains a critical challenge for safety and energy applications. Here, we report a fullerene-decorated PdCo nano-resistor network sensor that integrates nanostructuring, alloying, and surface-engineering approaches. The C_60_ layer enhances sensor performance by increasing the surface-to-volume ratio, enabling fast hydrogen diffusion, relieving mechanical stress during cycling, and guiding nanostructure morphology. Our composite device (20 nm C_60_/3 nm Teflon AF/5 nm Pd_63_Co_37_/30 nm Teflon AF) achieves a response time of 0.40 ± 0.06 s across 1–100 mbar H_2_ and detects 40 ppb H_2_ with a signal-to-noise ratio of 10 at room temperature. A poly(methyl methacrylate) (PMMA) topcoat further improves cycling stability and selectivity under 90% relative humidity and interfering gases. This design provides a scalable approach and opens the door to future adaptation of porous carbon-based frameworks and polymeric interlayers to realize robust, high-performance hydrogen sensors for real-world applications.

## Abstract (web summary)

Researchers have developed a fullerene-decorated PdCo nano-resistor network sensor that achieves fast hydrogen detection time of 0.40 seconds at 0.1% H_2_ and a detection limit of 40 ppb at room temperature.

## Introduction

Hydrogen plays a central role in clean energy technologies and industrial processes, yet its colorless, odorless nature and flammability require rapid and reliable detection systems^1–4^. The sensors must feature a response time (*t*_90_) of ≤1 s for H_2_ concentrations ranging from 0.1 to 10 vol.% (equivalent to H_2_ partial pressure of 1–100 mbar), a limit of detection (LOD) in the range of 1000 parts per million (ppm) for automotive and 10 parts per billion (ppb) for environmental monitoring applications^5,6^.

Recent advancements across both electrical and optical platforms in achieving ultra-fast response times and ppb-level sensitivity in hydrogen sensors have centered on nanostructured Pd hydride systems due to their high selectivity, excellent sensitivity, fast response times, and mechanical and chemical robustness (see Supplementary Table 1)^7–13^. Pd-based alloys (e.g., PdCo, PdAu) dominate high-impact designs. For instance, recent works on optical sensors by Nugroho et al. using scattering spectrum from PdAu nanoparticle@polymer nanocomposite materials^7^, and Luong et al. using transmission intensity through PdCo nanocap arrays^10^ explicitly demonstrate sub-second response with LODs around single-digit ppm. The LOD could be further improved to a few hundred ppb by making use of the particle swarm optimization algorithm to optimize the sensor array configurations^11^, neural-network-based data treatment to correct the baseline drift while testing in extreme humidified conditions^14^, or by simply stacking the sensors vertically to improve the signal-to-noise ratio (SNR)^12^. Compared to optical-based sensors, electrical transducing method shows great promise for achieving an ultra-sensitive LOD of a few ppb by virtue of the low noise signal readouts. Pham et al. proposed a PdCo composite hole array (CHA) nano-network with a measured LOD of 180 ppb; however, the response time fell short of the 1-s response time benchmark required for automotive applications^9^. Pd-doped oxides (e.g., Pd@Fe_2_O_3_ nanotubes) combine Pd’s catalytic activity with oxide heterojunctions, achieving 50 ppb LOD through synergistic surface reactions and p–n junction modulation^15^. Non-Pd materials, such as rGO/ZnO-SnO_2_ composites, also reach 50 ppb sensitivity via Pd doping and heterostructure engineering, though they lag behind Pd hydrides in speed and require high temperature operation of >300 °C^16^. These strategies highlight the critical role of alloying, nanostructuring, and hybrid material design tailoring for each transducing method and sensing mechanism in pushing the detection speed and sensitivity limits, with Pd-based systems remaining the gold standard for safety-critical applications. To our best knowledge, no single sensor reported to date has simultaneously achieved both ≤1-s response time and a ppb LOD operating at room temperature (Supplementary Fig. 1). Achieving ppb or sub-ppb detection limits not only benchmarks sensor performance under noise-limited conditions requiring high SNR and advanced signal processing^14,17^, but also enables critical applications such as trace hydrogen detection in deep-space missions^18^, the monitoring of low-abundance hydrogen isotopes like deuterium and tritium in nuclear environments^19^, and breath tests for assessing gastrointestinal health^20,21^.

In this study, we present a state-of-the-art hydrogen sensor based on a hexagonal nano-resistance network of Pd_x_Co_100-x_ alloy fabricated on a fullerene C_60_-coated glass substrate. This design incorporates four synergistic strategies to simultaneously achieve ultra-fast response and ppb sensitivity. First, the PdCo alloy was selected based on prior optimization studies demonstrating faster kinetics than pure Pd or Pd alloys such as PdAu and PdAg under identical structural and testing conditions^10^. In addition, the Co incorporation increases the strain-induced energy barrier to form the hydride effectively suppressing the hysteresis typically seen in pure Pd hydrides^10,22^. Second, sandwiching the C_60_ interlayer between the PdCo and the glass substrate, known to be inert to hydrogen chemisorption and physisorption at low pressures^23,24^, offers multiple functional benefits: (i) effectively doubles surface-to-volume ratio (SVR) of the sensing element thanks to the nanoporous structure that facilitates the hydrogen sorption processes through the C_60_ and PdCo interface^25^; (ii) it acts as a mechanical decoupler, enabling stress-free volumetric changes during hydrogenation cycles and enhancing device stability^26,27^; (iii) it serves as a morphological template, guiding the formation of the PdCo nanostructure; and (iv) it provides a chemically and mechanically robust foundation compatible with vacuum deposition^28^. Third, the nanostructuring strategy, combining nanosphere lithography (NSL) and glancing angle co-deposition (GLACD), produces a CHA structure with high SVR. This not only enhances hydrogen sorption kinetics^29^ but also amplifies resistance changes through increased electron scattering at grain boundaries and surfaces at the bottle-neck of the sensing elements^9^. In addition, the GLACD process further promotes alloy formation under far-from-equilibrium conditions, yielding defect-rich films with abundant active diffusion pathways^30^. Lastly, surface modification with Teflon AF (TAF) reduces the activation energy for hydrogen sorptions, accelerating response time while maintaining signal strength^7^. Together, these four integrated strategies enable a sensing platform that achieves sub-second response times and ppb-level hydrogen detection under ambient conditions.

Our results in vacuum-mode measurement demonstrate that 20 nm C_60_/5 nm Pd_63_Co_37_/30 nm TAF CHA sensors with a hole diameter of 450 ± 4 nm and a 500 nm array period achieve a response time of ≤0.8 s over a 1–100 mbar H_2_ pressure range, with a measured LOD of 144 ppb and an extrapolated LOD of 40  ppb. Remarkably, when the PdCo sensing layer is sandwiched between two TAF layers, the sensor can detect H_2_ as fast as 0.40 ± 0.06 s within the same pressure range, boasting the measured LOD down to 40 ppb with an exceptional SNR of 10. This C_60_/TAF/Pd_63_Co_37_/TAF stacked architecture holds promise for detecting hydrogen at single-digit ppb and sub-ppb levels. When coated with Poly(methyl methacrylate) (PMMA), the sensor demonstrates resilience against interference gases such as CO_2_, CH_4_, CO, maintains performance at up to 90% relative humidity (RH), and remains robust over hundreds of hydrogen cycling events using N_2_ as the carrier gas. This low-cost, lightweight, and energy-efficient platform offers a viable path toward meeting key performance metrics for automotive safety, concentration control, and environmental monitoring. These findings also highlight the broader potential of employing interlayers such as carbon-based materials, metal-organic frameworks and polymers with tunable gas permeability for designing next-generation hydrogen sensors.

## Results

**Fig. 1:**
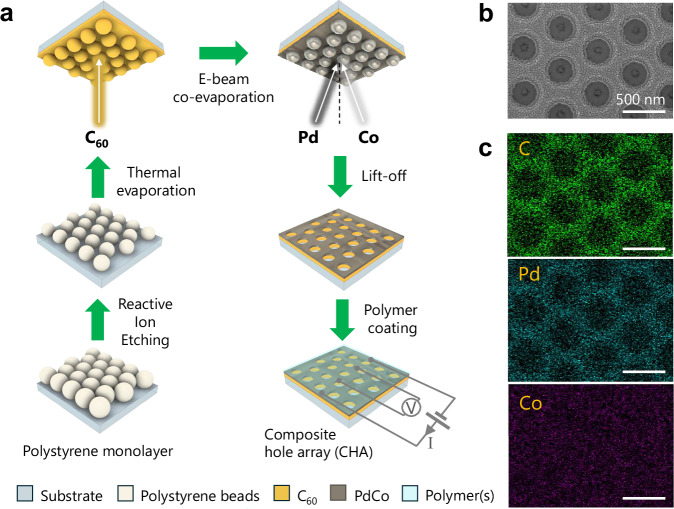
Fabrication scheme and morphology characterization. **a** Fabrication process of composite hole arrays (CHA). **b** A typical top-view SEM image and **c** EDS elemental maps of 20 nm C_60_/5 nm PdCo CHA_450_ based on weight percentages. The scale bars correspond to 500 nm. All microscopy images were obtained in triplicate (*N*  =  3) and display similar results.

The fabrication scheme of a PdCo CHA on fullerene C_60_-decorated glass substrate is depicted in Fig. 1a^9,31,32^, and is described in detail in the “Methods” section. First, a sacrificial nanosphere mask was prepared, and this involved depositing a monolayer of 500 ± 10 nm polystyrene (PS) nanospheres onto the substrates in a hexagonal close-packed arrangement. Subsequently, the size of the nanospheres in the monolayer was reduced via an oxygen plasma reactive ion etching (RIE) process by controlling the etching time *t*_RIE_. These substrates were then used as templates for C_60_ and PdCo alloy depositions in the later steps, and the resultant hole size of the CHA was determined by *t*_RIE_. Regarding the hydrogen sensing layer, we investigated a series of PdCo alloy compositions with palladium content ranging from 80% to 55%. Among these, the Pd_63_Co_37_ composition offered the best trade-off between response time and sensitivity^12^, and is therefore the primary focus of this study. A detailed discussion of this compositional optimization is provided in the later part of the article. The thicknesses of C_60_ and PdCo are specified in each case with the corresponding atomic force microscope (AFM) images. For brevity, we use the notations PdCo ${{{{\rm{CHA}}}}}_{{t}_{{{{\rm{RIE}}}}}}$ and C_60_/PdCo ${{{{\rm{CHA}}}}}_{{t}_{{{{\rm{RIE}}}}}}$ to refer to Pd_63_Co_37_ and C_60_/Pd_63_Co_37_ CHAs, with *t*_RIE_ representing the RIE etching time in seconds. Sensors coated with TAF (and PMMA) are labeled as C_60_/PdCo/TAF(/PMMA) ${{{{\rm{CHA}}}}}_{{t}_{{{{\rm{RIE}}}}}}$. The nano-architecture of the sensor was verified by a scanning electron microscopy (SEM) image of one of the devices (20 nm C_60_/5 nm PdCo CHA_450_) (Fig. 1b) and its atomic composition Pd_63_Co_37_ was confirmed by energy-dispersive spectroscopy (EDS) elemental mapping and line scans (Fig. 1c and Supplementary Fig. 2). The measured weight percentages of C, Pd, and Co were 79.5%, 15.4%, and 5.1%, respectively, corresponding to an atomic ratio of 63:37 between Pd and Co (see conversion Supplementary Table 3). The detailed experimental setups for the hydrogen sensing characterization were described in “Methods” and Supplementary Note 3. To mitigate contact and wire resistances, a four-point probe measurement technique was used throughout the paper (Fig. 1a).

**Fig. 2:**
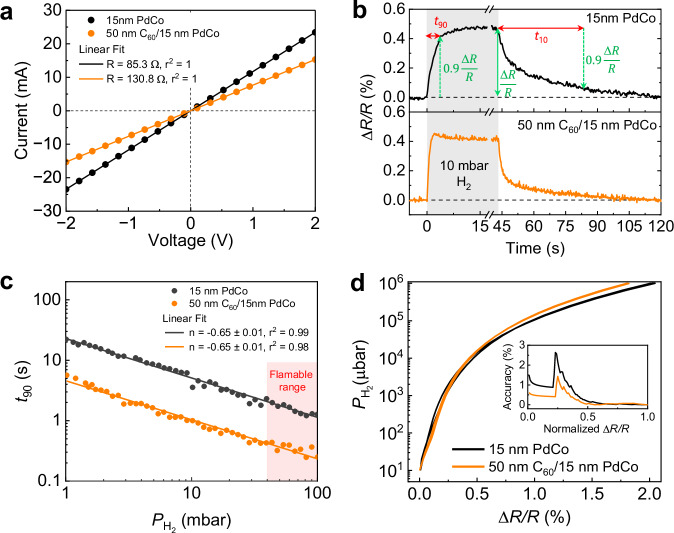
Sensing performances of 15 nm PdCo composite hole array CHA_450_ sensors with and without 50-nm C_60_ buffer layer. **a** Current-voltage (*I–V*) characteristics and resistances of the CHAs. **b** Sensors’ electrical resistance in response to a stepwise H_2_ pulse of ∼10 mbar (shaded areas). **c** Absorption time t_90_ of the sensors at ${P}_{{{{{\rm{H}}}}}_{2}}\,$= 1–100 mbar. **d** Electrical hydrogen sorption isotherms and sensor’s accuracy (inset) over ${P}_{{{{{\rm{H}}}}}_{2}}$ of 10^1^ to 10^6^ μbar. All measurements were performed in vacuum mode at room temperature. Source data are provided as a Source Data file.

To investigate the role of the buffer layer C_60_, we initially assessed the sensing performance of 15 nm PdCo CHA_450_ with and without 50 nm C_60_ underneath (Fig. 2). Both sensors show linear current-voltage (*I-V*) characteristics, in which the measured resistances (*R*) of the sensors with and without C_60_ in vacuum are 130.8 Ω and 85.3 Ω, respectively (Fig. 2a). The difference in *R* can be attributed to slight morphology variations in the bottle-neck regions of the two CHAs (Supplementary Fig. 7) and the interface differences between the substrate (glass or C_60_) and the PdCo layer. Figure 2b shows the sensors’ response to a step-wise H_2_ pulse of 10 mbar. Here, the percentage change in *R* or sensitivity of the electrical sensor as a function of ${P}_{{{{{\rm{H}}}}}_{2}}$ is defined as:

1 $$\frac{\Delta R}{R}\left(\%\right)=\frac{R\left({{{{\rm{H}}}}}_{2}\right)-R(0)}{R(0)}(\%)$$

where *R*(H_2_) and *R*(0) are resistances of the device with and without the presence of H_2_ gas, respectively. The hydrogen uptake of pure C_60_ at low H_2_ pressure and room temperature is negligible^23^, indicating that the change of *R* during (de)hydrogenation is primarily due to hydrogen sorption by Pd (Fig. 2b). The positive Δ*R*/*R* observed in both sensors indicates that electron scattering dominates the resistance change mechanism, as compared to hydrogen-induced lattice expansion mechanism during hydrogenation, which typically reduces sensor resistance^33,34^. In this case, more scattering centers from the absorbed hydrogen atoms at the interstitial sites were introduced to the system, causing the sensors’ resistance to increase. To quantify detection speed, absorption time (*t*_90_) and desorption time (*t*_10_) of the sensors at varying ${P}_{{{{{\rm{H}}}}}_{2}}$ are extracted from resistance dynamics as denoted in Fig. 2b. The commonly used *t*_90_ for the electrical sensors is defined as the elapsed time from the baseline resistance to 90% of the Δ*R*/*R* saturation, and *t*_10_ is defined as the elapsed time from the saturated level down to 10% of Δ*R*/*R*. The sorption kinetics of C_60_/PdCo CHA_450_ are noticeably accelerated as the *t*_90_ of the C_60_/PdCo sensor reduce by a factor of >4 over a pressure range of 1–100 mbar compared with those of PdCo sensor (Fig. 2c). In particular, *t*_90_ of C_60_/PdCo is only 5.6 s at ${P}_{{{{{\rm{H}}}}}_{2}}$ = 1 mbar, versus 24.7 s of PdCo. Remarkably, it takes ≤1 s for C_60_/PdCo CHA_450_ to detect ${P}_{{{{{\rm{H}}}}}_{2}}$ ≥ 10 mbar, which is lower than the lower limit of flammable H_2_ concentration in air (4 vol.% or 40 mbar). Additionally, the time taken for C_60_/PdCo sensor to release H_2_ across the same pressure range is more than twice as fast as that of the PdCo sensor (Supplementary Fig. 7). The acceleration in the sensor’s response time with the C_60_ interlayer may be attributed to: (i) the crystallographic structure of PdCo thin films on different substrates, or (ii) rapid H_2_ diffusion through the porous C_60_ walls in the CHA architecture, or (iii) a reduction in the activation energy barrier for H_2_ adsorption at the C_60_/PdCo interface. To gain insight into the contribution of C_60_ and examine the plausibility of each hypothesis, we conducted X-ray Diffraction (XRD) to analyze the crystal structure (Supplementary Fig. 10) and carried out a controlled experiment using PdCo sensors with a C_60_ top-coating (Supplementary Fig. 13). First, PdCo deposited on both glass and C_60_-coated substrates exhibits a similar crystallographic structure, with the dominant Bragg diffraction peak appearing at ~41.6° (Supplementary Fig. 10b), corresponding to the (111) orientation of the face-centered cubic (fcc) PdCo lattice^35^. The negligible differences in the lattice strain and lattice constant (Supplementary Table 4) make the first hypothesis unlikely. For the second hypothesis, a previous study by Samad et al. reported that the porosity of thermal-evaporated C_60_ thin film can exceed 50% when deposited at room temperature^36^. This aligns with the absence of distinct peaks in the XRD spectrum of C_60_ thin film (Supplementary Fig. 10a), confirming its amorphous nature and lack of long-range crystalline order of both the C_60_ film and the glass substrate^25,37^. Moreover, the nanohole structure opens up several new pathways for H_2_ access to PdCo sensing layer through the side walls of the C_60_. Any defect sites or metal-fullerene complexes that have micropores larger than the hydrogen molecules would facilitate the H_2_ permeation^23^. These combined properties allow hydrogen gas to access the PdCo transducer layer not only from the top surface, as in conventional sensor architectures, but also from the bottom interface via the underlying porous C_60_ film illustrated in Supplementary Fig. 8. This dual-sided accessibility increases effective gas exposure to the sensing material. The SVR is effectively doubled, reducing the average hydrogen diffusion path within the PdCo network and substantially improving the sensor’s response time compared to the sensor without C_60_ underneath. The third hypothesis is ruled out, as no enhancement in sensing performance was observed when a 20-nm C_60_ layer was deposited on top of a PdCo sensor (Supplementary Fig. 13). Thus, we can conclude that the enhancement of the response time is solely derived from the increase of the sensing element’s SVR by introducing a buffer layer C_60_. In Fig. 2c, the pressure-dependent sorption times of the C_60_/PdCo CHA_450_ sensor follow Sievert’s power-law, *t*_90_ ∝ ${\left({P}_{{{{{\rm{H}}}}}_{2}}\right)}^{{{{\rm{n}}}}}$^38^, as confirmed by a near-linear dependence between log(*t*_90_) and log(${P}_{{{{{\rm{H}}}}}_{2}}$) with a similar exponent (or the slope of the linear fit) *n* = −0.65 ± 0.01 as the PdCo CHA_450_, indicating similar sorption behavior or energy barrier for hydrogen adsorption/desorption in the two sensors, consistent with our previous conclusion. Figure 2d illustrates the sorption isotherms which present the sensitivity Δ*R*/*R* of each sensor across five orders of magnitude of ${P}_{{{{{\rm{H}}}}}_{2}}$, from 10^1^ to 10^6^ μbar, at room temperature. The hysteresis-free sensitivity of PdCo CHA_450_ is maintained in C_60_/PdCo CHA_450_, as confirmed by accuracy calculations (Fig. 2d inset and Supplemental Note 4). All sensors exhibit accurate readings with <3.0% uncertainty over a wide H_2_ pressure range thanks to the increase of the strain-induced energy barrier caused by the enhanced structural dislocation in the PdCo system^7,10,12^.

**Fig. 3:**
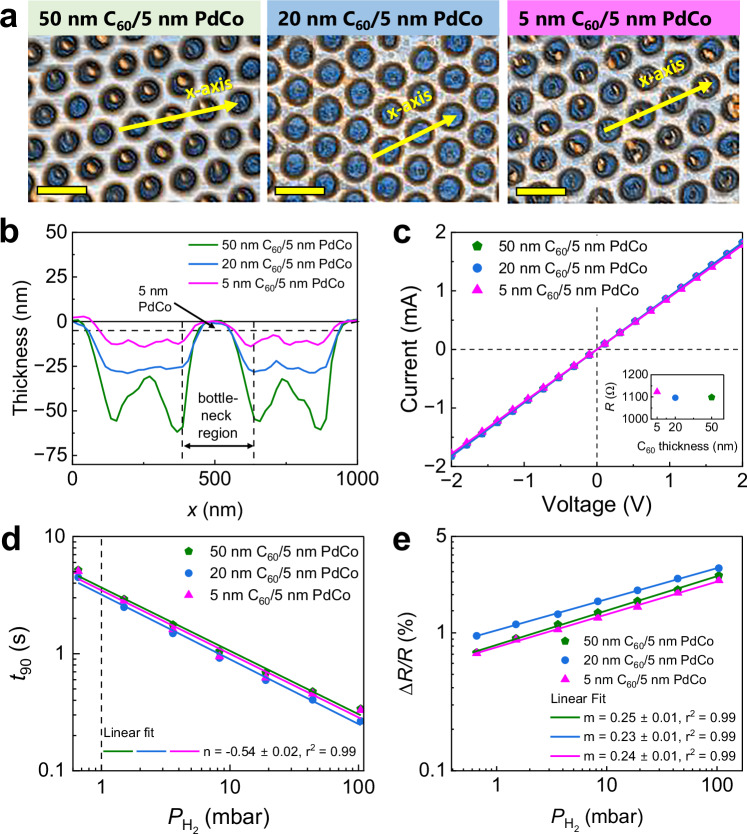
Morphology and sensing performances of C_60_/5 nm PdCo composite hole array CHA_450_ sensors with different C_60_ thicknesses. **a** AFM images of CHA_450_ sensors. The scale bars correspond to 500 nm. All microscopy images were obtained in triplicate (*N*  =  3) and display similar results. **b** The line profiles of the CHAs across bottleneck regions (along the *x*-axis denoted in figure a). The profiles are shifted in such a way that the top surface of the CHA is zero. **c** Current-voltage (*I–V*) characteristic of the sensors measured in vacuum and calculated resistances (inset). **d** Extracted absorption time *t*_90_ and **e** sensitivity Δ*R/R* of the sensors at ${P}_{{{{{\rm{H}}}}}_{2}}$ = 0.7–105 mbar. All measurements were performed in vacuum mode at room temperature. Source data are provided as a Source Data file.

Next, the dependence of CHA_450_’s sensing response on the thickness of the C_60_ layer was studied (Fig. 3). In this case, 5-nm-nominal thick of Pd_63_Co_37_ was utilized as a sensing layer and was deposited on substrates coated with 5-nm, 20-nm, and 50-nm thick of C_60_. Figure 3a shows typical AFM images of C_60_/5 nm PdCo CHA_450_ sensors etched at *t*_RIE_ = 450 s, resulting in an average hole diameter of *D*_hole_ = 350 ± 5 nm (extracted from the line profiles of the AFM images, Fig. 3b). It is worth noting that the polystyrene remnants from the PS beads, left at the center of the holes after the lift-off process, did not interfere with the electrical signals of the devices. The baseline resistances of C_60_/5 nm PdCo CHA_450_ sensors were obtained from the linear *I-V* characteristics (Fig. 3c), with an average resistance of *R* = 1100 ± 10 Ω. The C_60_/5 nm PdCo CHA_450_ sensors showed a slight improvement in response time and sensitivity (*t*_90_ < 3.7 s and Δ*R*/*R* > 0.8% at ${P}_{{{{{\rm{H}}}}}_{2}}$ = 1 mbar) compared to C_60_/15 nm PdCo CHA sensor, by virtue of the enhancement of reaction kinetics through nanostructuring^29^. Interestingly, CHA_450_ sensors with different C_60_ thicknesses exhibited comparable *t*_90_ and *t*_10_ values (Fig. 3d and Supplementary Fig. 12). These findings further support our assertion that hydrogen diffusion through C_60_ is rapid and not rate-limited in our nano hole-array structure (with a few hundred nm of the hydrogen diffusion path in C_60_). It is feasible to locally probe the diffusion rate of the C_60_/PdCo bilayer using optical techniques^39^ and extract the response times at different locations on the films such as near the edges or at the center, such analysis is beyond the scope of the present study. Among the three CHA_450_ sensors tested, the 20 nm C_60_/5 nm PdCo CHA_450_ sensor exhibited the highest sensitivity, which is required for achieving a low LOD. As a result, in the following sensor design, we will maintain the nominal thicknesses of C_60_ and PdCo layers at 20 nm and 5 nm, respectively, while varying *t*_RIE_ to achieve a smaller feature size and, consequently, a larger SVR – a key factor for ultra-fast sensing responses.

### Hole size-dependent sensing performance

**Fig. 4:**
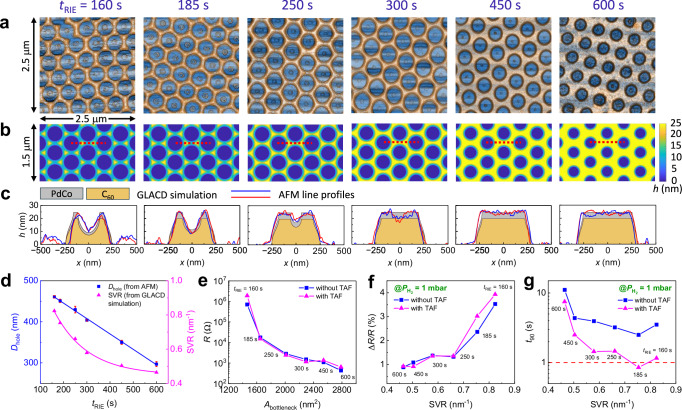
Morphologies and sensing performances of 20 nm C_60_/5 nm PdCo composite hole array ${{{{\rm{CHA}}}}}_{{t}_{{{{\rm{RIE}}}}}}$. **a** Top-view AFM images of ${{{{\rm{CHA}}}}}_{{t}_{{{{\rm{RIE}}}}}}$ and **b** the corresponding simulated morphologies of CHAs with different etching times. All microscopy images were obtained in triplicate (*N*  =  3) and display similar results. **c** Cross-sectional views from glancing angle co-deposition GLACD simulations along the dashed red lines in b, showcasing the simulated thickness of each layer, in comparison with AFM line profiles. **d** Estimated hole diameters from AFM averaged from 10 holes (data are presented as mean values +/- SD) and calculated surface-to-volume ratio SVR of PdCo layer from GLACD simulations. **e** Resistances of the 20 nm C_60_/5 nm PdCo ${{{{\rm{CHA}}}}}_{{t}_{{{{\rm{RIE}}}}}}$ sensors with and without Teflon AF (TAF) coating as a function of the bottleneck cross-sectional area *A*_bottleneck_. **f** Response time *t*_90_ and **g** sensitivity of the sensors at ${P}_{{{{{\rm{H}}}}}_{2}}$ = 1 mbar as a function of SVR. Source data are provided as a Source Data file.

In the combined NSL and GLACD method^10^, the nanohole diameters (*D*_hole_) or the sizes of the sensing network elements, especially the bottle-neck feature sizes, can be controlled by adjusting the etching time *t*_RIE_ of the PS beads (Fig. 4). Figure 4a depicts the top-view AFM images of CHAs with varying *t*_RIE_ from 600 s to 160 s. When *t*_RIE_ < 160 s (Supplementary Fig. 14), a nanotriangle array was achieved instead of a nanohole array, resulting in a discontinuous nano-network. Additionally, an in-house GLACD simulation code with MATLAB^40–42^ was utilized to model the morphology and thickness of each layer (C_60_ and PdCo) in each ${{{{\rm{CHA}}}}}_{{t}_{{{{\rm{RIE}}}}}}$ sensor (see Supplementary Note 7), with the results shown in Fig. 4b, c. The cross-sectional view from GLACD simulations in Fig. 4c aligns well with AFM line profiles, confirming that the C_60_ layer is mechanically stable enough to resist penetration by high-energy metal atoms during deposition. Moreover, C_60_ functions as a critical morphological template, effectively shaping the geometry of the resulting PdCo nanostructures. Morphologically, the hole diameter decreases linearly with *t*_RIE_ (Fig. 4d). At smaller *t*_RIE_ values (<300 s), where the bead size after etching exceeds 400 nm, the shadow effect becomes more pronounced, resulting in a U-shaped indentation in the middle of the bottle-neck region, leading to a reduction in the total thickness in this region. The simulated cross-sections along and across the bottleneck region of ${{{{\rm{CHA}}}}}_{{t}_{{{{\rm{RIE}}}}}}$, along with the thicknesses of PdCo layer (*h*_PdCo_) projected on a flat surface, are presented in Supplementary Fig. 15. In some regions, the estimated *h*_PdCo_ can be as thin as 2 nm, and the actual *h*_PdCo_ might be even thinner than the simulation suggests (Fig. 4c). Moreover, the SVR of the PdCo layer is significantly enhanced as *t*_RIE_ decreases, due to PdCo being deposited in narrower gaps between the PS beads and on the undulating C_60_ surface. The detailed SVR calculations are presented in Supplementary Note 7.2, and all parameters of 20 nm C_60_/5 nm PdCo ${{{{\rm{CHA}}}}}_{{t}_{{{{\rm{RIE}}}}}}$ are summarized in Supplementary Table 5. In particular, CHA_185_ has an SVR of ~0.75 nm^−1^, which is 1.5 times larger than that of CHA_450_ (0.5 nm^−1^).

The changes in morphology significantly influence the resistances and the sensing performance of the devices, as illustrated in Fig. 4e–g. As the hole diameter increases, the feature size of the resistor network decreases, leading to higher resistance. Figure 4d, e shows that the sensor’s resistance increases slightly when *D*_hole_ increases from 310 nm to 410 nm. In this range, the thickness of the network element does not change, as depicted in Fig. 4c. Therefore, the slight increase in resistance is attributed to the reduction in sensor bottleneck width. However, when *D*_hole_ increases from 410 nm to 460 nm, a U-shaped indentation forms, causing the network’s resistance to increase by nearly three orders of magnitude. A closer inspection reveals that the thickness of the triangular regions (the ends of the resistance network element, see Supplementary Fig. 16) does not change with *D*_hole_, keeping the resistivity nearly constant in those regions. Consequently, the total resistance of the network is dominated by the resistance at the bottleneck, *R*_*b*_, due to the reduction in thickness because of the shadowing effect as seen in Fig. 4c. The response of the electric current during (de)hydrogenation primarily depends on hydrogen sorption kinetics at the bottleneck^9^. The measured baseline *R* of ${{{{\rm{CHA}}}}}_{{t}_{{{{\rm{RIE}}}}}}$ is plotted against the bottleneck’s cross-sectional area *A*_bottleneck_ of the PdCo layer (Fig. 4e). Overall, the *R* of the nano-resistor network significantly increases as the *A*_bottleneck_ decreases. This nonlinear relationship was observed in several electrical resistance studies of ultra-thin films at a typical thickness of ~10 nm^43–46^, and could be explained by three thickness-dependent resistivity models: (i) the Fuchs–Sondheimer model for surface and interface scatterings^47^, (ii) the Mayadas–Shatzkes model for grain boundary scattering^46^, and (iii) the Namba model considering the ratio of surface roughness to the electron mean free path^48^. Given the complexity of the sensors’ structure and the ultra-thin sensing layer, all these scattering mechanisms may be present and influence electron transport. Lacy developed a more general resistivity model, incorporating scatterings from surface, grain boundaries, and surface roughness into a single parameter *η*, known as the thickness reduction factor^49^. The theoretical model best fits our experimental data when *η* = 1.5 nm for the ultra-thin film regime (*t*_RIE_ ≤ 300 s), and *η* = 0.9 nm for the uniform thickness regime (*t*_RIE_ ≥ 300 s) (see Supplementary Note 7.3 for detailed fitting). The fitting suggests that these scattering effects are more pronounced in the U-shaped region due to the smaller grain boundaries in a thinner PdCo region as shown in SEM images in Supplementary Fig. 16.

All sensors underwent sensing response measurements, with sorption times and sensitivity across ${P}_{{{{{\rm{H}}}}}_{2}}$ ranging from 100 to 1 mbar being extracted (Supplementary Note 7.4). In order to find the optimal sensor design, *t*_90_ and Δ*R/R* at ${P}_{{{{{\rm{H}}}}}_{2}}$ = 1 mbar of each sensor are depicted in Fig. 4f, g (labeled in blue filled squares). The sensitivity is seen proportional to the SVR of the PdCo layer, as a larger SVR increases the number of available Pd sites for hydrogen adsorption and dissociation^50^. Additionally, response time improves at smaller film thickness, as the hydrogen diffusion path to the interstitial sites of the small PdCo nanocrystalline domains is reduced^29^. Among the sensors, the CHA_185_ sensor exhibits the fastest response time of 2.5 s while maintaining a high sensitivity of ~2.5% at 1 mbar of H_2_. However, this still falls short of the 1-s response time benchmark required for automotive applications^6^. We explored increasing the Co composition in the alloy and reducing the thickness of the PdCo layer to improve the sensor’s response time. Nonetheless, a 5-nm Pd_63_Co_37_ layer emerged as the best compromise between high sensitivity and rapid response as a higher composition of Co (e.g., 45%) significantly reduces the hydrogen solubility of the sensing element and a thinner nominal deposited thickness (e.g., 3.5 nm) might result in discontinuous resistance network with slower response times (Supplementary Fig. 20).

Perfluorinated polymers, such as Polytetrafluoroethylene (PTFE)^7,51,52^ and TAF^12^, have been widely used as a coating layer for H_2_ sensors due to their proven ability to effectively boost the H_2_ sorption kinetics by lowering the activation energy for both H_2_ absorption and desorption. Therefore, 30 nm of TAF was then thermally evaporated on all 20 nm C_60_/5 nm PdCo ${{{{\rm{CHA}}}}}_{{{{{\rm{t}}}}}_{{{{\rm{RIE}}}}}}$ sensors. Compared to uncoated sensors (labeled in blue filled squares), TAF-coated sensors (labeled in pink filled triangles) display a comparable baseline resistance, a similar rise in Δ*R/R*, and a reduction in *t*_90_ with the increase in SVR as shown in Fig. 4e, f. Notably, the TAF coating led to a significant improvement in response time. At ${P}_{{{{{\rm{H}}}}}_{2}}$ = 1 mbar, the *t*_90_ of each TAF-coated ${{{{\rm{CHA}}}}}_{{{{{\rm{t}}}}}_{{{{\rm{RIE}}}}}}$ sensor decreased by ~2 s (Fig. 4g), approaching the 1-s benchmark. In fact, the CHA_185_/TAF sensor surpassed this benchmark, achieving an ultra-fast *t*_90_ of <0.8 s. Interestingly, in both TAF-coated and uncoated data revealed a kink in the *t*_90_ curves at *t*_RIE_ = 185 s. Although the CHA_160_ sensor had a higher SVR, its *t*_90_ was slower than that of CHA_185_ sensor, likely due to the formation of defects, such as vacancies and dislocations, in the few-nanometer-thick film, which could increase the energy barrier for hydride formation^53^.

**Fig. 5:**
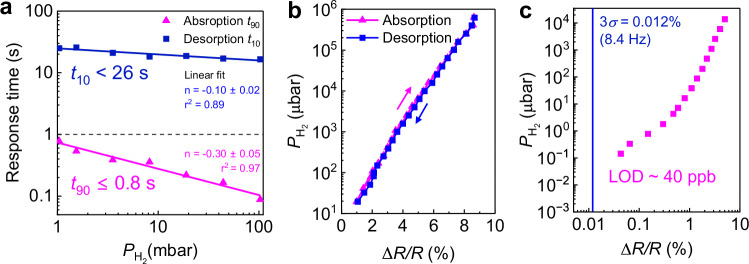
Sensing performances of 20 nm C_60_/5 nm PdCo/30 nm Teflon AF (TAF) composite hole array CHA_185_. **a** Absorption time (*t*_90_) and desorption time (*t*_10_) of the sensor in response to varying ${P}_{{{{{\rm{H}}}}}_{2}}$ from 100 to 1 mbar extracted from Supplementary Fig. 21a. **b** Sensor’s hydrogen sorption isotherms across five orders of ${P}_{{{{{\rm{H}}}}}_{2}}$ from 10^1^ to 10^6^ µbar. **c** Δ*R/R* plot as a function of ${P}_{{{{{\rm{H}}}}}_{2}}$ extracted from Supplementary Fig. 21b. The vertical blue line denotes the defined LOD of 3*σ* ≈ 0.012% at 8.4 Hz sampling frequency (Supplementary Note 8). All measurements were performed in vacuum mode at room temperature. Source data are provided as a Source Data file.

The 20 nm C_60_/5 nm PdCo/30 nm TAF CHA_185_ sensor was found to be optimal and its detailed sensing performance is presented in Fig. 5. In Supplementary Fig. 21a, the normalized sorption kinetics of the sensor in response to ${P}_{{{{{\rm{H}}}}}_{2}}$ ranging from 100–1 mbar exhibit a superior SNR, with the noise of the acquired signal *σ* = 0.006% at 12.2 Hz sampling frequency (see Supplementary Note 8), enabling reliable extraction of the response time. Remarkably, the sorption times increase at a much slower rate (with *n*_absorption_ ≈ −0.30 and *n*_desorption_ ≈ −0.10) as ${P}_{{{{{\rm{H}}}}}_{2}}$ decreases (Fig. 5a). Particularly, *t*_90_ remains ≤0.8 s in the 1–100 mbar ${P}_{{{{{\rm{H}}}}}_{2}\,}$ range, much faster than any Pd-based electrical hydrogen sensors measured under similar conditions (see Supplementary Table 2). The sensor releases H_2_ in less than 26 s within the tested ${P}_{{{{{\rm{H}}}}}_{2}}$ range. In addition, the sorption isotherms of the sensor summarized in Fig. 5b show a hysteresis-free characteristic. To further examine its detection capability, the sensor was tested in diluted H_2_ gas in an N_2_ balance (Supplementary Fig. 21b). Although C_60_ is reported to be high permeable to N_2_^54^, the sensor’s signal remains unaffected by the N_2_ pressure variation (see Supplementary Fig. 22a) and the interdiffusion of N_2_ with H_2_ has a negligible effect on the sensing performance under vacuum mode measurement within the tested ${P}_{{{{{\rm{H}}}}}_{2}}$ range as illustrated in Supplementary Fig. 23. Thus, H_2_ and N_2_ mixture can be utilized in vacuum mode measurement to reliable extract the sensitivity of the sensor at low partial H_2_ pressures. The result in Fig. 5c shows that the C_60_/PdCo/TAF CHA_185_ sensor can reliably resolve the hydrogen pressure as low as 144 μbar (≈144 ppb). By defining the LOD as the ${P}_{{{{{\rm{H}}}}}_{2}}$ at which the detectable resistance change signal = 3*σ*, where *σ* = 0.004% at 8.4 Hz sampling frequency (see Supplementary Note 8), extrapolating the sensitivity obtained from Fig. 5c, the TAF-coated CHA_185_ sensor is able to detect the ${P}_{{{{{\rm{H}}}}}_{2}}$ of 0.04 µbar (≈40 ppb), approaching the DOE requirement of 10 ppb LOD for environmental monitoring applications^5^.

**Fig. 6:**
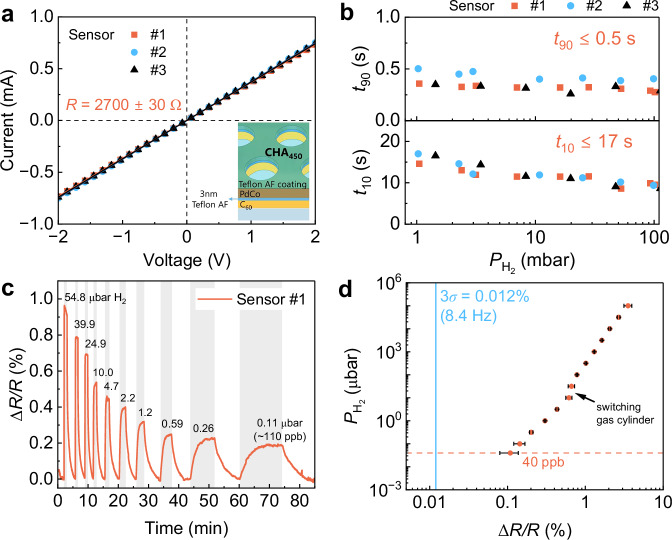
Sensing performances of 20 nm C_60_/3 nm Teflon AF/5 nm PdCo/30 nm Teflon AF composite hole array CHA_450_ sensors. **a** Current-voltage (*I–V*) characteristics of 3 sensors with the same device structure (inset), **b** absorption time (*t*_90_) and desorption time (*t*_10_) of the 3 sensors in 1–100 mbar H_2_ pressure range. **c** Δ*R/R* response of sensor #1 upon partial H_2_ pressures of 54.8–0.11 µbar measured at 8.4 Hz sampling frequency. **d** Averaged Δ*R/R* plot as a function of ${P}_{{{{{\rm{H}}}}}_{2}}$ with the error bars indicate the standard deviation from 3 devices. Data are presented as mean values +/- SD. The vertical blue line denotes the defined LOD of 3*σ* ≈ 0.012% at 8.4 Hz sampling frequency (Supplementary Note 8). All measurements were performed in vacuum mode at room temperature. Source data are provided as a Source Data file.

Given the significant enhancement in H_2_ sorption kinetics provided by the TAF top coverage, one might wonder why the C_60_ layer is not entirely replaced by TAF. The fabrication process of the sensor involves the lift-off of PS beads to create the hole array structure. However, TAF does not adhere to the glass substrate as well as C_60_ to survive the lift-off. Despite varying etching times, no conductivity was detected from the TAF/PdCo/TAF CHA sensors. This may stem from the porous nature of the TAF polymer and from the penetration of Pd and Co atoms into the soft TAF layer, both of which can lead to a PdCo thin film with numerous pinholes (see Supplementary Fig. 9) and to discontinuities in the PdCo thin films^55^. Consequently, instead of replacing the entire C_60_ layer with TAF, a very thin layer of TAF was deposited on top of C_60_, followed by PdCo co-deposition and another TAF coating, as depicted in Fig. 6a inset. The characterizations of three sensors with the same stacking structure of 20 nm C_60_/3 nm TAF/5 nm PdCo/30 nm TAF CHA_450_ are shown in Fig. 6. All sensors exhibit linear *I-V* characteristics (Fig. 6a), from which the electric power was extracted and showed in Supplementary Fig. 24. At 0.1 mA of applied source current, the sensor consumes a negligible power of 25 μW or 90  mWh. The average resistance of the sensor on the C_60_/TAF-coated substrate is *R* = 2700 ± 30 Ω, approximately twice that of the sensor on the C_60_-coated substrate. This increase can also be attributed to the slight influence of the TAF’s porosity and the penetration of Co and Pd atoms into the ultrathin soft TAF layer, whereas PdCo forms a smoother layer on C_60_ (Supplementary Fig. 9). Here, we demonstrate the hydrogen sensing performance of the CHA_450_-based sensor only because these pinhole effects also prevent the formation of a continuous PdCo nano-network in hole array structures with smaller channels, such as CHA_185_ or CHA_250_ (Fig. 4a). All three tested devices exhibited significantly faster response and release times, with record fast *t*_90_ = 0.40 ± 0.06 s and *t*_10_ = 16 ± 1 s across the H_2_ pressure range of 1–100 mbar as shown in Fig. 6b, the fastest sensor ever reported in the literature at a similar operation conditions (see Supplementary Table 2). The improved sensing response of samples with TAF underneath can be attributed to a combination of reduced grain size observed in the XRD analysis (Supplementary Table 4), efficient gas permeation through the underlayer, and a lowered energy barrier for hydrogen sorption at the TAF/PdCo interfaces^7^. Figure 6c demonstrates the detection capability of one of the C_60_/TAF/PdCo/TAF CHA_450_ sensors through step-wise pressure pulses of diluted H_2_ in an N_2_ balance, corresponding to partial H_2_ pressures ranging from 54.8 to 0.4 µbar. The sensor effectively detected the lowest ${P}_{{{{{\rm{H}}}}}_{2}}$ = 0.11 µbar (~110 ppb) with an impressive sensitivity of 0.2%. Notably, the signal is clearly distinct from the background noise (*σ* = 0.004% at *f*_sampling_ = 8.4 Hz) and is not affected by the N_2_ gas carrier pressure (Supplementary Fig. 22b), which is crucial for precise monitoring. The LOD was then confirmed by repeating the measurements for sensors #2 and #3 (Supplementary Fig. 27). The sensitivity averaged from 3 individual sensors across six orders of hydrogen pressures, as shown in Fig. 6d, suggests a reliable detection of 40 ppb with sensitivity of 0.11 ± 0.03% and an extrapolated LOD as low as 100 part-per-trillion (ppt) (Supplementary Fig. 27d). Our sensor’s exceptional performance ranks it among the fastest and most sensitive hydrogen sensors currently available (Supplementary Note 1).

### Stability and selectivity tests

**Fig. 7:**
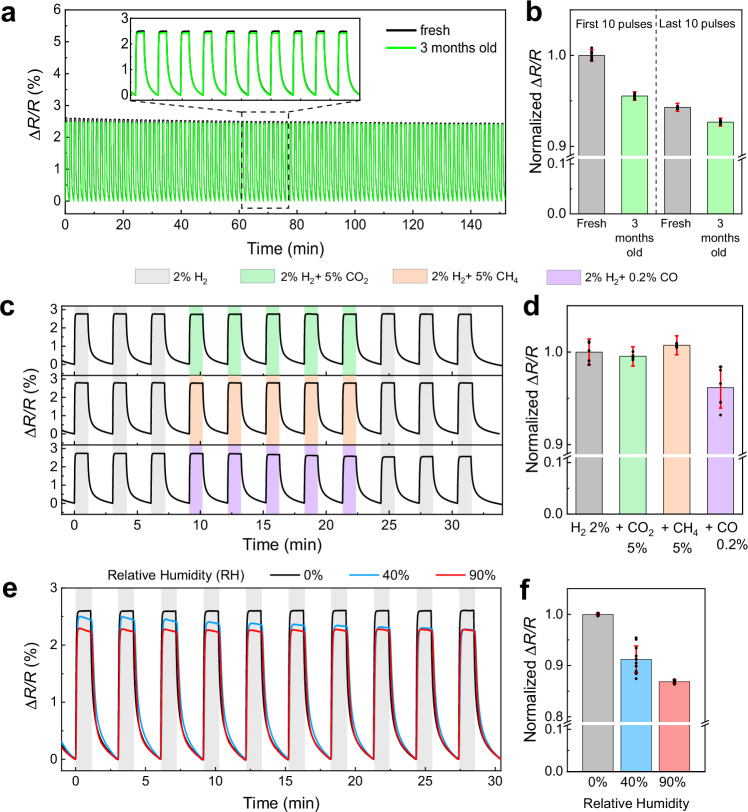
Aging and selectivity tests of 20 nm C_60_/5 nm PdCo/30 nm Teflon AF/PMMA composite hole array CHA_450_ sensors. **a** Δ*R/R* responses of fresh and 3-month-old samples upon 100 cycles (30 s/60 s of loading/unloading) of 2% H_2_ balanced in N_2_ with a total flow of 400 ml/min. **b** Normalized sensor signal of the first and last 10 pulses in figure a. The error bars denote the standard deviation from 10 cycles. **c** Time-resolved Δ*R/R* response of the sensor to 3 pulses of 2% H_2_ followed by 5 pulses of 2% H_2_ + 5% CO_2_, 2% H_2_ + 5% CH_4_, 2% H_2_ + 0.2% CO; then followed by 3 pulses of 2% H_2_. **d** Normalized sensor signal to the one obtained with 2% H_2_ in N_2_ flow. The error bars denote the standard deviation from 5 cycles. **e** Time-resolved Δ*R/R* response of the sensor to 10 pulses of 2% H_2_ with different relative humidity (RH) and **f** normalized signal to the one obtained with 2% H_2_ in dry condition. The error bars denote the standard deviation from 10 cycles. Data in Fig. 7b, d, f are presented as mean values +/- SD. All measurements were performed at room temperature, using N_2_ as carrier gas. Source data are provided as a Source Data file.

In addition to response time and the detection limit, the sensor’s capability to reliably detect H_2_ under the influence of aging, toxic gases, humidity and temperature is crucial for practical application. While TAF-coated sensors improve hydrogen sorption kinetics, their poor selectivity leaves them vulnerable to degradation in the presence of toxic gases like CO or extreme humidified condition (see Supplementary Fig. 29)^7,12^. To overcome this limitation, a high H_2_ permeable poly(methyl methacrylate) (PMMA) has been utilized as an additional protective layer (Fig. 7)^7,9,10,56^. It is worth noting that a 50-nm thick PMMA coating layer shows negligible effect on the sensors’ response times thanks to its high permeability and diffusivity to H_2_ (Supplementary Fig. 30)^57^. First, we assessed the stability of both a fresh and a 3-month-old (stored in a N_2_ filled glovebox) 20 nm C_60_/5 nm PdCo/30 nm TAF/PMMA CHA_450_ sensor upon 100 cycles of loading/ unloading 2% H_2_ in N_2_ using a flow mode set-up (see Methods and Supplementary Note 3). Overall, the sensitivity of the sensors remained stable throughout the cycling test, with a small variation of <5% (Fig. 7a, b). The 3-month-old sensor showed a comparable (de)absorption time to the fresh one (Fig. 7a inset), demonstrating resilience against mechanical stress and strain from repeated lattice expansion and contraction. It can be ascribed to the high degree of rotational disorder of the C_60_ molecules around one of their central axes at room temperature^27,58^, which allows the PdCo film to expand and contract with minimal mechanical constraint. Even if chemical bonding occurs at the PdCo/C_60_ interface (see X-Ray Photoelectron Spectroscopy (XPS) analysis in Supplementary Fig. 11), this molecular mobility ensures that the sensor retains mechanical flexibility and stability, mitigating the degradation due to the buckle or global detachment of the PdCo film from the substrate in clamped films^59–61^.

Next, we conducted tests to evaluate the sensor resistance to poisonous gases and humidity. In deactivation tests, the sensor was exposed to 5% of CO_2_, 5% of CH_4_, and 0.2% of CO, each mixed with 2% H_2_ in N_2_, respectively. As shown in Fig. 7c, d, the normalized sensitivity remained within ±4%, well below the ±5% accuracy required for hydrogen sensors^6^. The response time remained almost unchanged (within 1 s) before and after exposure to 5% CO_2_ or 5% CH_4_, though a larger fluctuation of ~3 s in absorption time was observed in high concentrations of CO (0.2%) (Supplementary Fig. 31). It is important to note that 0.2% of CO represents an extreme testing condition, as typical urban CO levels are around 10 ppm (~0.001%)^62^. We also performed humidity tests with gas carriers at 40% and 90% relative humidity (RH) compared to the dry condition (Fig. 7e, f). The sensor’s sensitivity gradually decreases after each cycle due to water vapor condensation on the sensing elements, which can hinder hydrogen adsorption and dissociation^63,64^. Δ*R/R* at 40% RH appears to converge with the signal at 90% RH, suggesting that the effect of humidity may saturate once a certain amount of water is deposited on the sensor surface. The average signal amplitudes in 40% RH and 90% RH were 91 ± 3% and 87 ± 1%, respectively, compared to the dry reference environment. Notably, the impact of humidity is temporary since the water molecules are weakly bound to the sensor surface and can be cleared by injecting dry air^65^. The sensor’s performance recovered after a few pulses of dry 2% H_2_ in N_2_ (Supplementary Fig. 32). This result places the C_60_/PdCo/TAF/PMMA CHA sensor among the top-performing hydrogen sensors under extremely humidified conditions (RH = 90%)^8,14^. While this study focuses on idealized conditions, we acknowledge that evaluating sensor performance in air is essential for real-world applications. Some preliminary results with polyvinyl alcohol (PVOH)-coated sensors show that oxygen interference can be mitigated; however, the coating introduces trade-offs in hydrogen diffusion and limits applicability under humid conditions^11,66^. Further optimization of coating thickness and tandem architecture to balance selectivity, response time, and environmental robustness remains an important future direction beyond the scope of this work.

To address the temperature stability of the system and the temperature dependence of resistance, we evaluated sensor performance of one of our CHAs over a range from room temperature to 115 °C and consistently observed hydrogen response (see Supplementary Fig. 33). However, two notable effects emerged at elevated temperatures: (i) increased baseline drift, which may result from thermally induced morphological changes in the nanoparticle network such as expansion or coalescence that alter percolation pathways, and (ii) a significant drop in sensitivity, consistent with suppressed β-phase hydride formation and enhanced hydrogen desorption kinetics^8^. These findings indicate that room temperature offers the best balance between baseline stability and sensing performance for our PdCo nano-network structure.

## Discussion

In summary, we demonstrate a PdCo nano-resistor hydrogen sensor enhanced with a fullerene C_60_ interlayer, which combines nanostructuring, alloying, and surface/interface engineering strategies. The incorporation of C_60_ improves performance by increasing the active surface area of the sensing element, facilitating hydrogen transport, mitigating stress during cycling, and directing the nanostructure formation. This unique and high-throughput design simultaneously achieves ultra-fast and ultra-low H_2_ detection. In one CHA_185_ configuration (*D*_hole_ = 450 ± 4 nm) consisting of a stacking 20 nm C_60_/5 nm Pd_63_Co_37_/30 nm TAF, the sensor demonstrates a response time of ≤0.8 s over a hydrogen pressure range of 1–100 mbar, achieving a LOD of ~144 ppb. In another CHA_450_ configuration (*D*_hole_ = 350 ± 5 nm) with TAF on both sides the sensing element PdCo (20 nm C_60_/3 nm TAF/5 nm Pd_63_Co_37_/30 nm TAF), the sensor can detect H_2_ as fast as 0.40 ± 0.06 s within the same pressure range, boasting an exceptional measured LOD down to 40 ppb level. The addition of a PMMA coating provides excellent selectivity against interfering gases (CO_2_, CH_4_, CO) and ensures stable performance under up to 90% relative humidity with N_2_ as a gas carrier. The sensor also maintains high sensitivity after extensive cycling and prolonged storage under inert conditions. It is noteworthy that the high-performance sensors were fabricated using simple, scalable methods that are inherently compatible with standard semiconductor processes, making the design a strong candidate for industrial integration. A detailed discussion of the fabrication steps, scalability, and potential challenges related to large-area uniformity, material cost, integration, and long-term stability is provided in Supplementary Note 10. Our CHA architecture with an interfacial layer of C_60_ and TAF demonstrates strong potential to satisfy the demanding performance and stability requirements of hydrogen sensing in both automotive and environmental monitoring applications. More broadly, this materials design framework, which emphasizes scalable, permeable, and stable interlayers, can guide future strategies for engineering surface and interface layers (e.g., using carbon-based^67^, metal–organic frameworks^68^ and polymers^61^) across diverse chemical sensing platforms.

## Methods

### Materials

Polystyrene (PS) nanospheres (Polysciences Inc., average bead diameter *D* = 500 ± 10 nm, CV = 2%) and ethanol (Sigma-Aldrich, 98%) were used to fabricate the nanosphere monolayers. Sulfuric Acid (Lab Alley, 93%) and hydrogen peroxide (Lab Alley, 30%) were utilized for the petri dish and substrate cleaning processes. Polymethyl methacrylate (PMMA), fullerene C_60_ (99.9%), and acetone (≥99.5%) were purchased from Sigma-Aldrich. Teflon AF 2400 (TAF) was obtained from Dupont. Palladium (99.95%), and cobalt (99.95%) from Kurt. J Lesker Company were utilized for electron beam depositions. Deionized (DI) water (18 MΩ.cm) was used for all experiments.

### Composite nanohole array (CHA) fabrication

The composite nanohole array (CHA) fabrication started with the growing of hexagonal close-packed nanosphere monolayers on glass substrates or silicon wafers using the air/water interface method^31,69,70^. First, a 14-cm diameter petri dish was cleaned with piranha solution (4:1 mixture of sulfuric acid and hydrogen peroxide) and subsequently filled with 22 ml of DI water. Concurrently, a separate colloidal suspension was prepared by mixing 300 μl of the stock PS nanosphere solution, 1000 μl of DI water, and 650 μl of ethanol. This suspension was continuously dispensed via a syringe pump (kdScientific, series 100) at 0.012 mL/min onto the water-filled petri dish. The nanospheres were allowed to self-assemble for ~3 h until the monolayer formed large domains covering ~75% of the water surface. Substrates were then carefully slid horizontally beneath the floating monolayer. Finally, excess water was drained and the coated substrates were then left to dry overnight. The nanosphere diameter *D* = 500 nm was chosen since the monolayers can have the highest quality with this size. The PS monolayer, then, went under the RIE process using Trion Phantom III RIE Etcher (40  mTorr O_2_, ICP power = 25 W, RF power = 10 W). The etching times were adjusted from 600 s to 150 s for the bead size reduction. In the next step, fullerene C_60_ was thermally evaporated on top of the templates with the deposition rate of 0.26 Å/s, followed by electron beam co-deposition of Pd and Co with 0.05-nm/s total deposition rate yielding a thin Pd_63_Co_37_ alloy film. To increase the mixing uniformity between Pd and Co, the sample holder was rotated azimuthally with a constant rotation rate of 80 rpm during the deposition process. The substrate was then cut into a standard size of 10 × 5 mm^2^ followed by monolayer lift-off by using the scotch tape technique. In some cases, the device was coated by a layer of TAF and a PMMA film. For TAF coating, the TAF powder was thermally evaporated at the rate of 0.02 nm/s to form a uniform coating with a nominal thickness of ~30 nm^12^. For PMMA coating, PMMA powder was mixed in Acetone at 10 mg/mL concentration at 80 °C until fully dissolved. The solution was then cooled down to room temperature before being spin-casted on top of the hole array at a speed of 5000 rpm for 120 s, followed by the final soft bake at 85 °C for 20 min. The approximate thickness of the coating PMMA layer is 50 nm^10^.

### Morphology, composition and crystal structure characterization

The nanomorphologies of fabricated samples were measured by an AFM (Park NX10), and the AFM images were then analyzed using XEI - Image Processing and Analysis Software and Gwyddion^71^.

SEM was performed with a SU-9000 system, Hitachi (resolution of 0.4 nm at 20 kV). EDS elemental mapping was performed with 150 mm Oxford XMaxN detector.

XRD patterns were measured using a powder diffractometer (XRD, Rigaku) with Cu-Kα radiation (λ = 1.54056 Å, 40 kV, and 40 mA). The range of 2 theta angle from 39° to 44° with speed 0.1 degree per minute and 0.03° for the sampling width for all thin film samples on the glass substrate.

XPS spectra of thin films on Si (110) wafer were taken using Thermo Scientific Nexsa G2 XPS system with Al Kα radiation (1486.6 eV) and the line width of 0.25−0.30 eV.

### Sensing characterization

The sample was mounted in a home-built H_2_ gas cell^70^. The resistance of the CHAs was measured by a collinear 4-point probe using a SourceMeter KEITHLEY 2635B as a constant current source. In vacuum mode measurement, H_2_ gas pressure was monitored by three independent pressure transducers with different ranges which cover the pressure range of 2.7E-6 to 1.1 bar (two PX409-USBH, Omega and a Baratron, MKS). By using diluted 4% or 100 ppm or 10 ppm H_2_ balanced in N_2,_ we can control the partial H_2_ pressure as low as 40 μbar (~40 ppm) or 100 nbar (~100 ppb) or 10 nbar (~10 ppb), respectively. In flow mode measurement, 4% H_2_ in nitrogen mixture gases (Airgas) were diluted with ultra-high purity nitrogen gas from Airgas company to targeted concentrations by a commercial gas blender (GB-103, MCQ Instruments). The gas flow rate was kept constant at 400 ml/min at 1 atm for all measurements. All experiments were performed at room temperature if not stated otherwise.

### Abridged disclaimer

The view expressed herein do not necessarily represent the view of the U.S. Department of Energy or the United States Government.

### Reporting summary

Further information on research design is available in the Nature Portfolio Reporting Summary linked to this article.

## Supplementary information

Supplementary Information

Peer Review File

Reporting Summary

## Source data

Source Data

## Data Availability

Source data are provided with this paper. Additional information can be obtained from the corresponding authors upon request. Source data are provided with this paper.
