# Supplementary material for: Fullerene-decorated PdCo nano-resistor network hydrogen sensors with sub-second response and parts-per-billion detection at room temperature
Source: Nat Commun. 2025 Dec 19;17:977. doi: 10.1038/s41467-025-67708-2 (PMC12847907; doi:10.1038/s41467-025-67708-2)
Supplement: Supplementary file 1 — Supplementary Information [file 41467_2025_67708_MOESM1_ESM.pdf]

Supplementary Information

for

Fullerene-decorated PdCo nano-resistor network hydrogen sensors with sub-second response and parts-per-billion detection at room temperature

Tu Anh Ngo[1]*, Ashwin T. Magar[1], Minh T. Pham[1], Hoang M. Luong[1,2], Thi Thu Trinh Phan[3], M. Tuan Trinh[3], Michael Jung[4], George K. Larsen[5], Yiping Zhao[1], and Tho D. Nguyen[1]*

[1] Department of Physics and Astronomy, University of Georgia, Athens, Georgia 30602, USA.

[2] Department of Electrical Engineering, Faculty of Engineering, Chulalongkorn University, Bangkok 10330, Thailand.

[3] Department of Chemistry and Biochemistry, Utah State University, Logan, 84322, USA.

[4] School of Environmental, Civil, Agricultural and Mechanical Engineering, University of Georgia, Athens, Georgia 30602, USA.

[5] Hydrogen Isotope Science Group, Savannah River National Laboratory, Aiken, South Carolina 29808, USA.

* Correspondence to T.A.N. (anhngo@uga.edu) or to T.D.N. (ngtho@uga.edu).

Table of Contents

Supplementary Note 1. State-of-the-art Pd-based H_2 sensors 3

Supplementary Note 2. SEM and EDS elemental mapping images 7

Supplementary Note 3. Hydrogen sensing characterization setups 8

Supplementary Note 4. Sensing characteristics of PdCo CHAs with and without C_{60} interlayer 11

Supplementary Note 5. AFM, XRD and XPS spectra of PdCo thin films 13

Supplementary Note 6. Sensing characteristics of CHAs on different C_{60} thicknesses 18

Supplementary Note 7. CHAs with different etching time t_{RIE} 19

Supplementary Note 7.1. Morphology characterization and glancing angle deposition (GLACD) simulation 19

Supplementary Note 7.2. Surface-to-volume (SVR) ratio calculation 22

Supplementary Note 7.3. Resistivity model 24

Supplementary Note 7.4. Sensing characteristics of CHAs with different etching time t_{RIE} 26

Supplementary Note 8. Noise evaluation 33

Supplementary Note 9. Stability, interference gases and humidity tests 34

Supplementary Note 10. Scalability, feasibility, and potential challenges 37

Supplementary references 39

Supplementary Note 1. State-of-the-art Pd-based H_2 sensors

Supplementary Table 1. Ultra-sensitive state-of-the-art H_2 sensors with LOD < 10 ppm reported for the last 10 years.

Ref.	Transducing method	Material and structure	Measurement condition	t_{90} (s) at 0.1%†	t_{10} (s) at 0.1%†	Hysteresis free?	LOD (ppb)	RH test range	T (°C)	Interference gas test
The U.S. DoE's requirements for environmental monitoring applications				<30 s (600 ppb)	<30 s (600 ppb)	Yes	10 ppb	0% – 98%	-30 – 80	O_2, CO, CO_2, hydrocarbons
The U.S. DoE's requirements for automotive applications				<1 s (0.1%)	<1 s (0.1%)	Yes	0.10%	0% – 98%	-30 – 80	O_2, CO, CO_2, hydrocarbons
This work	Electrical	C_{60}/PdCo/Teflon AF/PMMA CHA_{185}	Vacuum & flow mode in N_2	0.8	26	Yes	144	0% - 90%	RT	CO, CO_2, CH_4
This work	Electrical	C_{60}/Teflon AF/ PdCo/Teflon AF/PMMA CHA_{450}	Vacuum	<0.5	17		40			
[3]Nature Electronics 2025	Electrical	DPP-DTT thin film on Pt electrode	Flow mode in Air	0.84	6.63	N.A.	192	15% - 80%	20 - 120	EtOH, Me_2CO, MeOH, Toluene
[4]Scientific Reports 2024	Electrical	Polyaniline PANI hollow nanotube	Flow mode in N_2	15 (1 ppm)	17 (1 ppm)	N.A.	1000	N.A.	N.A.	N.A.
[5]ACS Sensors 2024	Electrical	Pd-Doped α-Fe_2O_3 Nanotubes	Flow mode in Air	49 (200 ppm)	533 (200 ppm)	N.A.	50 (300° C)	0% - 90%	300	C_3H_6O, NH_3, NO_2, C_2H_6O, CO, CH_4, CO_2, SO_2, H_2S
[6]Nano Energy 2023	Magneto-optical	$Pd_{67}Co_{33}$/ Teflon AF nanopatches	Vacuum & flow mode in N_2	0.4	2.8	Yes	1000	0% - 90%	N.A.	CO, CO_2, CH_4
[7]Light: Science & Applications 2023	Opto-electrical	Platinum–silicon nanojunctions	Flow mode in Air	10 (1.3 ppm)	N.A.	N.A.	1000*	N.A.	RT	Air
[8]Microsystems & Nano-engineering 2023	Thermo-electric	Pt nanoparticles @Al_2O_3 on P+/N+ single-crystalline silicon thermopiles	Flow mode in Air	1.9	1.4	N.A.	1000 (120° C)	N.A.	50 - 120	CH_4, C_2H_6, Acetone, Toluene, CO, EtOH
[9]Microsystems & Nano-engineering 2022	Electrical	Pd-doped rGO/ZnO-SnO_2	N.A.	4 (100 ppm)	8 (100 ppm)	N.A.	50 (380° C)	N.A.	380	HCHO, C_4H_{10}, C_7H_8, CO_2

Ref.	Transdu-cing method	Material and structure	Measure-ment condition	t_{90} (s) at 0.1%†	t_{10} (s) at 0.1%†	Hyster-esis free?	LOD (ppb)	RH test ran-ge	T (°C)	Interfer-ence gas test
[10]Nature Commun-ications 2022	Optical	Pd nanoparticles @PMMA	Flow mode in Air	40 mins (250 ppb)	50 mins (250 ppb)	N.A.	250	N.A.	RT	Air
[11]Nature Commun-ications 2021	Optical	$Pd_{80}Co_{20}$/ Teflon AF nanopatches	Vacuum & flow mode in Air	0.85	N.A.	Yes	2500	0% - 40%	20 - 42	CO, CO_2, CH_4
[12]Sensors and Actuators B: Chemical 2021	Electrical	Pd@Ni foam	Flow mode in N_2	138 (2%)	300 (2%)	N.A.	7	0% - 99.5 %	30 - 70	O_2, He, Ar, CO_2, Co, NH_3, CH_4, C_2H_4
[13]ACS Applied Nano Materials 2021	Electrical	PdCo nanohole arrays	Vacuum & flow mode in N_2	10.8	20	Yes	180	0% - 90%	RT	Air, CO
[14]Carbon 2021	Electrical	Pd nanoparticles on Y_2O_3/carbon nanotubes	Flow mode in N_2	N.A.	N.A.	N.A.	90 ppb (RT) 5 ppb (100° C)	0% - 75%	100	CO, NO_2, H_2S
[15]Nature Materials 2019	Optical	PdAu nanoparticles @PTFE/ PMMA	Vacuum & flow mode in Air	1	5	Yes	1000 (in Ar) 5000 (in Air)	N.A.	30 - 60	CO_2, CH_4, CO, NO_2, Air
[16]IEEE 2015	Electrical	Multiwalled carbon nanotubes	Vacuum & flow mode in Air	75 (60 ppb)	N.A.	N.A.	400	N.A.	RT	CO, NH_3, CH_4, H_2S, NO_2, and acetone
[17] Nanosale 2015	Electrical	Pd nanoparticles @CPPy	Flow mode: H_2 (abs.), 9 N_2:1 O_2 mixture (des.)	4.5 (20 ppm)	27 (20 ppm)	N.A.	100	N.A.	RT	N.A.
[18] Scientific Reports 2015	Electrical	Pd nanoflower/gra phene	Flow mode in N2	80 (10 ppm)	N.A.	N.A.	100	N.A.	RT	N.A.

†If not specified otherwise, *Extrapolated data, N.A. = not addressed.

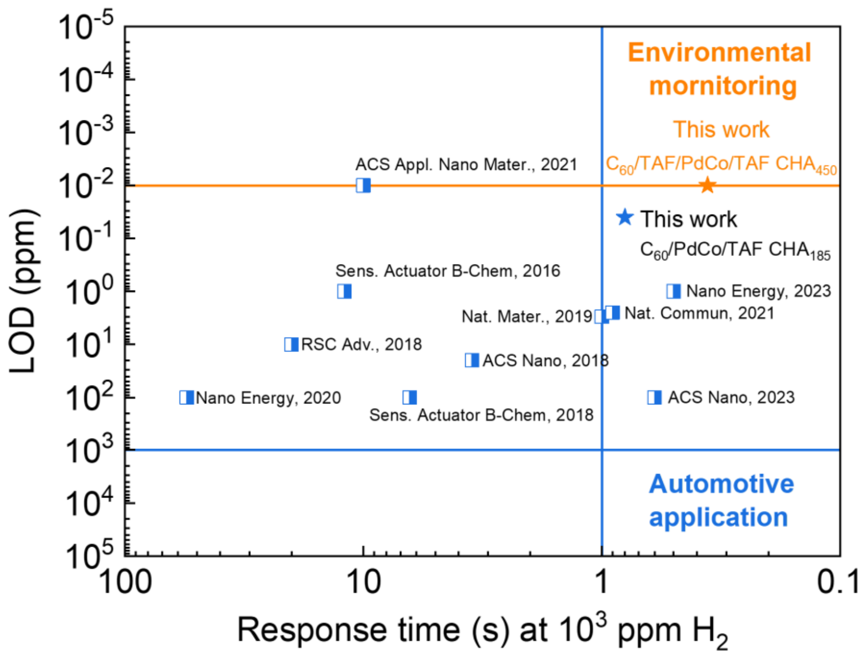

Supplementary Figure 1. State-of-the-art Pd-based hydrogen gas sensors' sensing metrics. The response time and LOD requirements for automotive[1] and environmental monitoring[2] applications are denoted in red and green lines, respectively.

Sens. Actuator B-Chem, 2016	Ref. [19]	Nano Energy, 2020	Ref. [20]
RSC Adv., 2018	Ref. [21]	ACS Appl. Nano Mater., 2021	Ref. [13]
ACS Nano, 2018	Ref. [22]	Nat. Commun, 2021	Ref. [23]
Sens. Actuator B-Chem, 2018	Ref. [24]	Nano Energy, 2023	Ref. [6]
Nat. Mater., 2019	Ref. [15]	ACS Nano, 2023	Ref. [25]

Supplementary Table 2. Sensing metrics of electrical H_2 gas sensors operating at room temperature with either response time < 30 s at 1 mbar (or 0.1 vol.%) H_2 or LOD < 1000 ppm. N.A. = not addressed.

**Device structure**	**Response time (s) (t_{90} if not specified)**	**Ambient pressure (mbar)**	**LOD (ppm)**	**Ref.**
C_{60}/PdCo/Teflon AF/PMMA CHA_{185}	≤ 0.8	1	0.144	This work
C_{60}/Teflon AF/ PdCo/Teflon AF CHA_{450}	≤ 0.4	1	0.04	This work
PMMA-coated Pd-Co nanohole array	10.8	1	0.180	13
Hollow Pd nanotube network (PVA@Pd)	2.1	1	10	26
Pd-Ni alloy thin films	$t_{63} = 5$	10	N.A.	27
Palladium nanowire engineered nanofiltration	13	1	1000	28
Ultrasmall grained Pd nanopattern	12	30	2.5	29
Pd-capped Mg film	6	10	1	30
Pd@Au core-shell nanoparticles	15	200	1000	31
Networks of ultrasmall palladium nanowires	~25	1	N.A.	32
Pd-decorated silicon nanomesh	$t_{80} \sim 10$	1	50	33
SiO_2 nanorod coated-Pd	17	10	10	34
Pt-TiO_2	10 ± 5	1	30	35
Polyurethane@Pd	24	1	20	36
Pd nanowires	25	1	50	37
Pd NP/graphene	300	1	20	38
PdNi Nanogap	0.5	20	500	39
Discontinuous palladium films on Polyimide	5	40	5000	40
Pd nanorod	7	N.A.	1000	41
Pd nanoparticles	1.2	10	10^4	42
Pd nanoparticles coated Multi-Walled Carbon Nano Tubes	15	300	N.A.	43

Supplementary Note 2. SEM and EDS elemental mapping images

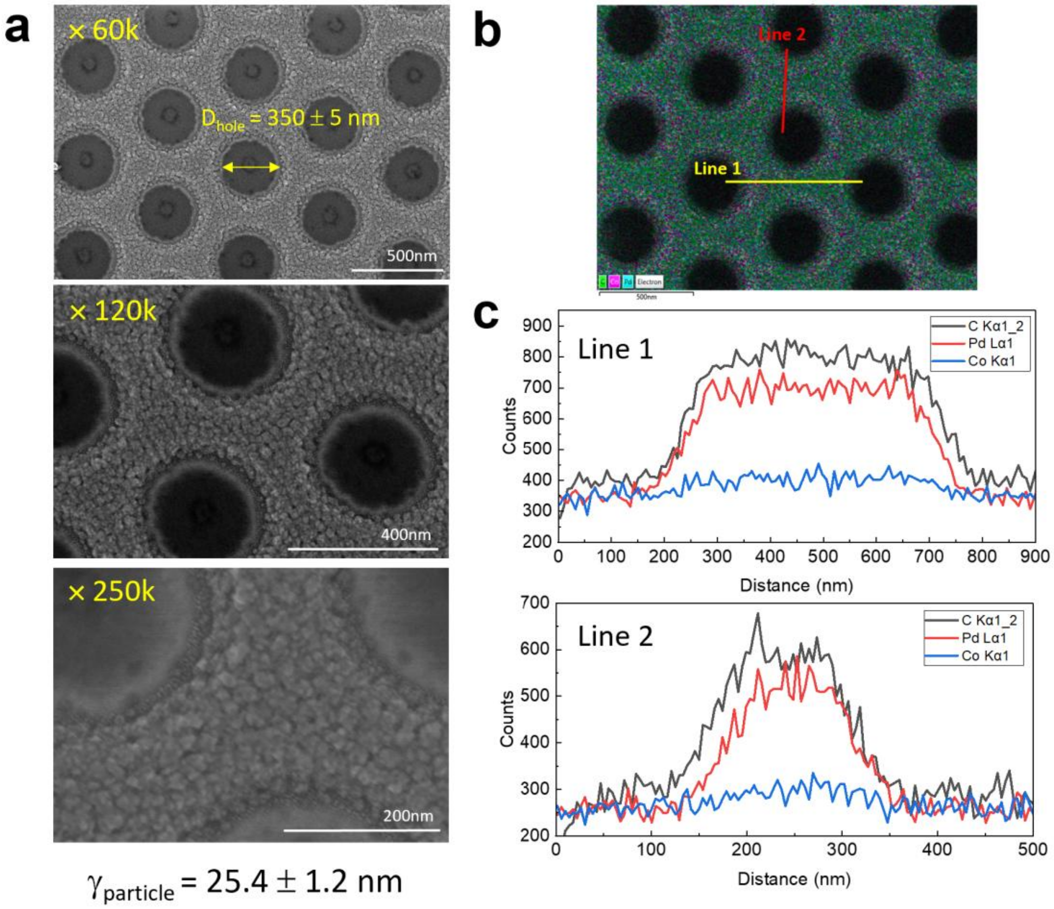

Supplementary Figure 2. a Scanning electron microscopy (SEM) images of 20 nm C_{60}/5 nm PdCo CHA_{450} (hole diameter D_{hole} = 350 ± 5 nm) at different magnifications. The estimated diameter of the PdCo particles on the surface is $\gamma_{particle}$ = 25.4 ± 1.2 nm. **b** Energy-dispersive spectroscopy (EDS) elemental layered mapping. **c** EDS line spectra along two lines denoted in (b). Counts are based on weight percentages. All microscopy images were obtained in triplicate (N = 3) and display similar results. Source data are provided as a Source Data file.

Supplementary Table 3. Weight % to atomic % conversion table

Elements	Map (Supplementary Fig. 2b)		Line 1 (Supplementary Fig. 2c)		Line 2 (Supplementary Fig. 2c)	
	Wt. %	At. %	Wt. %	At. %	Wt. %	At. %
**C**	79.5		7.20 ± 0.10		9.30 ± 0.10	
**Pd**	15.4	63	0.87 ± 0.02	67 ± 6	0.58 ± 0.02	63 ± 7
**Co**	5.1	37	0.24 ± 0.02	33 ± 3	0.19 ± 0.02	37 ± 4

Supplementary Note 3. Hydrogen sensing characterization setups

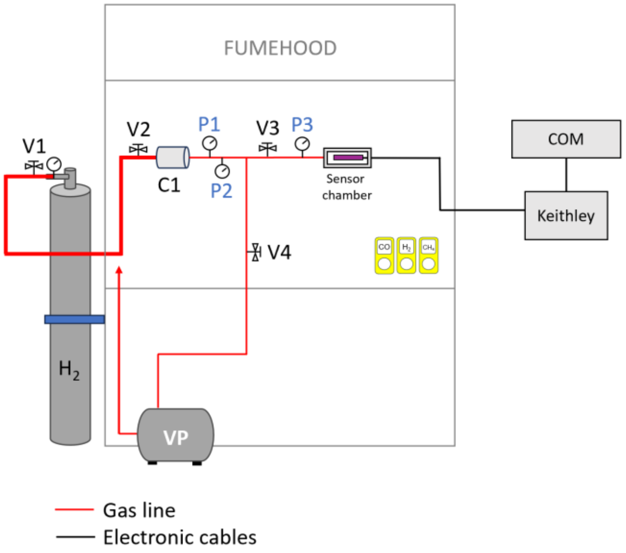

Supplementary Figure 3. H_2 electrical sensing vacuum mode setup.

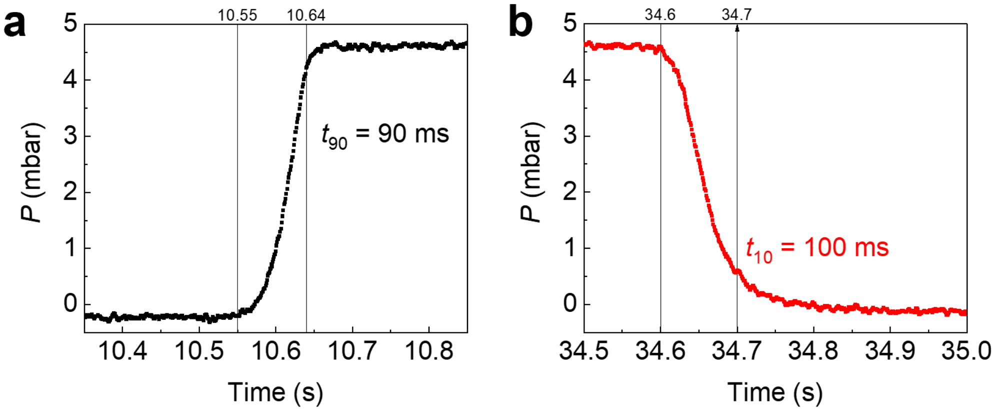

Supplementary Figure 4. Reaction time of the pressure transducers while **a** loading and **b** unloading H_2 in vacuum mode measurement.

The vacuum mode set up is depicted in Supplementary Fig. 3. Different H_2 gas pressure in the sensor chamber can be prepared by recurringly diluting pure H_2 gas or the gas mixture of 4% H_2 in N_2 (Airgas) from chamber 1 (C1) to the sensor chamber by several gas valves (V1-V3). The H_2 pressures are monitored by three independent pressure transducers (two PX409-USBH, Omega and a Baratron, MKS). The pressure transducers exhibit a reaction time of 1 ms. Our system

requires 90 and 100 ms to attain equilibrium pressure during loading and unloading, respectively (see Supplementary Fig. 4). These timeframes were not excluded from the response time calculation. Finally, the chamber sensor is isolated to C1, and the gas inside the chamber is pumped out using valve V4 to achieve the base pressure of ~5 x 10^{-4} mbar in the chamber. The resistance of the sensors is recorded during the process using 4-point probe meaurement by a Keithley 2635B current source. In order to further exploring the measurement at a lower H_2 pressure, 4% or 100 ppm or 10 ppm of H_2 mixed gas in N_2 balance can be used. In this case, we can prepare a mixture gas with H_2 pressure in sensor chamber down to 1 mbar, which is equivalent to partial hydrogen pressure/concentration of 40 ppm or 100 ppb or 10 ppb, respectively.

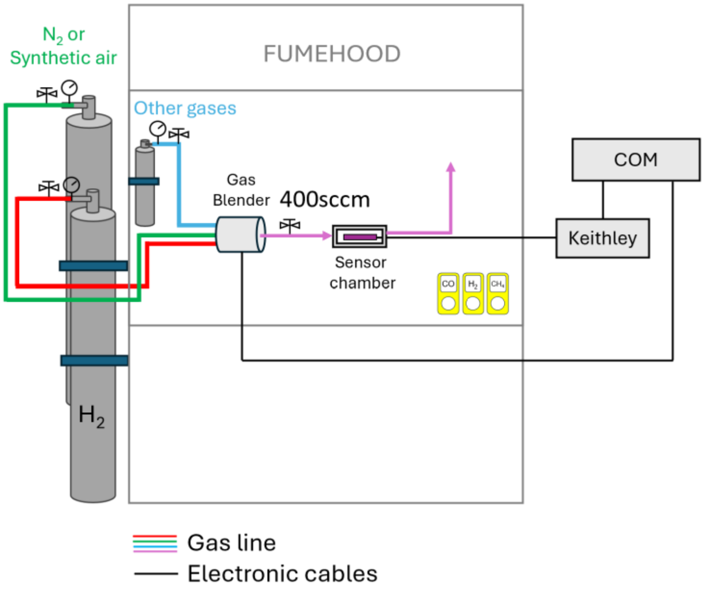

Supplementary Figure 5. H_2 electrical sensing flow mode setup.

In addition to the vacuum mode setup, the gas sensing measurement is also performed in flow mode to mimic the leakage (Supplementary Fig. 5). 4% H_2 balance in N_2 are further diluted with ultra-high purity N_2 gas to the targeted concentrations by a commercial gas blender (GB-103,

MCQ Instruments). The gas flow rate is kept constant at 400 ml/min or 400 sccm at 1 atm for all measurements. The gas cell and gas outlet are placed inside a fume hood during the measurement.

The flow mode measurement is essential for practical validation, however, the response time in flow mode is significantly affected flow rate since external diffusion becomes the rate-limiting step.[44] In contrast, vacuum mode eliminates these transport limitations as the gas pressure reaches equilibrium in < 100 ms (Supplementary Fig. 4) and allows us to isolate the internal transport through the layered structure. Therefore, while flow mode better reflects real-world deployment conditions for hydrogen sensors, vacuum mode offers valuable insights into the material-level optimization and can serve as a benchmark for assessing the limitations imposed by sensor housing and gas delivery design. It raises important considerations for future device engineering, such as enhancing gas dynamics via integrated pumps, or flow channel optimization.

Supplementary Note 4. Sensing characteristics of PdCo CHAs with and without C_{60} interlayer

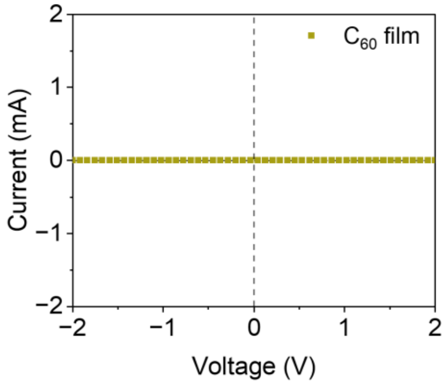

Supplementary Figure 6. Curren-voltage (I-V) characteristics of C_{60} film.

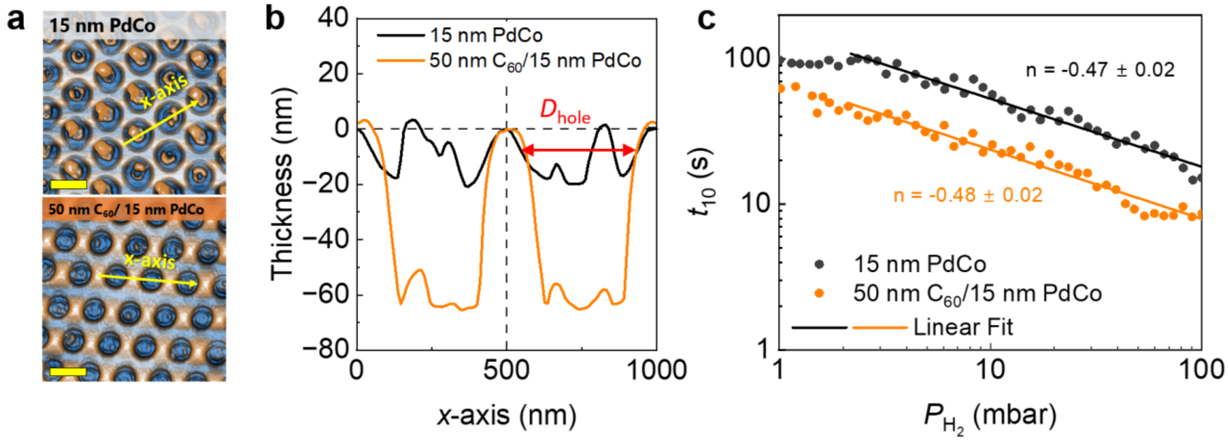

Supplementary Figure 7. **a** Atomic force microscope (AFM) images of the CHA_{450}'s with and without 50-nm C_{60} interlayer. The scale bars correspond to 500 nm. **b** The line profiles of the CHAs along the x-axis denoted in figure a. The profiles are shifted in such a way that the top surface of the CHA is zero. The polystyrene remnants from the PS beads, left at the center of the holes after the lift-off process, did not interfere with the electrical signals of the devices, however it might affect the line profile of the 15 nm PdCo CHA_{450} sensor. **c** Desorption time t_{10} of the sensors at $P_{H_2} = 1 – 100$ mbar. All microscopy images were obtained in triplicate (N = 3) and display similar results. Source data are provided as a Source Data file.

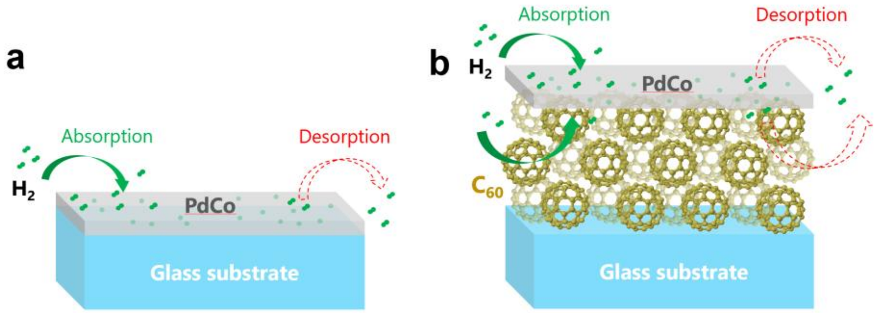

Supplementary Figure 8. Schematic illustration comparing hydrogen absorption and desorption pathways in PdCo thin films on **a** bare glass substrate and **b** C_{60}-coated substrate. C_{60} molecule illustration was adapted from virtual Chemistry 3D by romuald.poteau_at_univ-tlse3.fr, licensed under CC BY 4.0.

The sensor accuracy is calculated by the following equation[45]:

$$\text{Accuracy} = \frac{|\log(P_{\text{Abs}}) - \log(P_{\text{Des}})| \times 100}{|\log(P_{\text{Abs}}) + \log(P_{\text{Des}})|/2}\ (\%), \quad \text{(S1)}$$

where P_{Abs} and P_{Des} are the pressures reading (in μbar) during hydrogen absorption and desorption, respectively, extracted from the isotherm curves (Fig. 2d main text).

Supplementary Note 5. AFM, XRD and XPS spectra of PdCo thin films

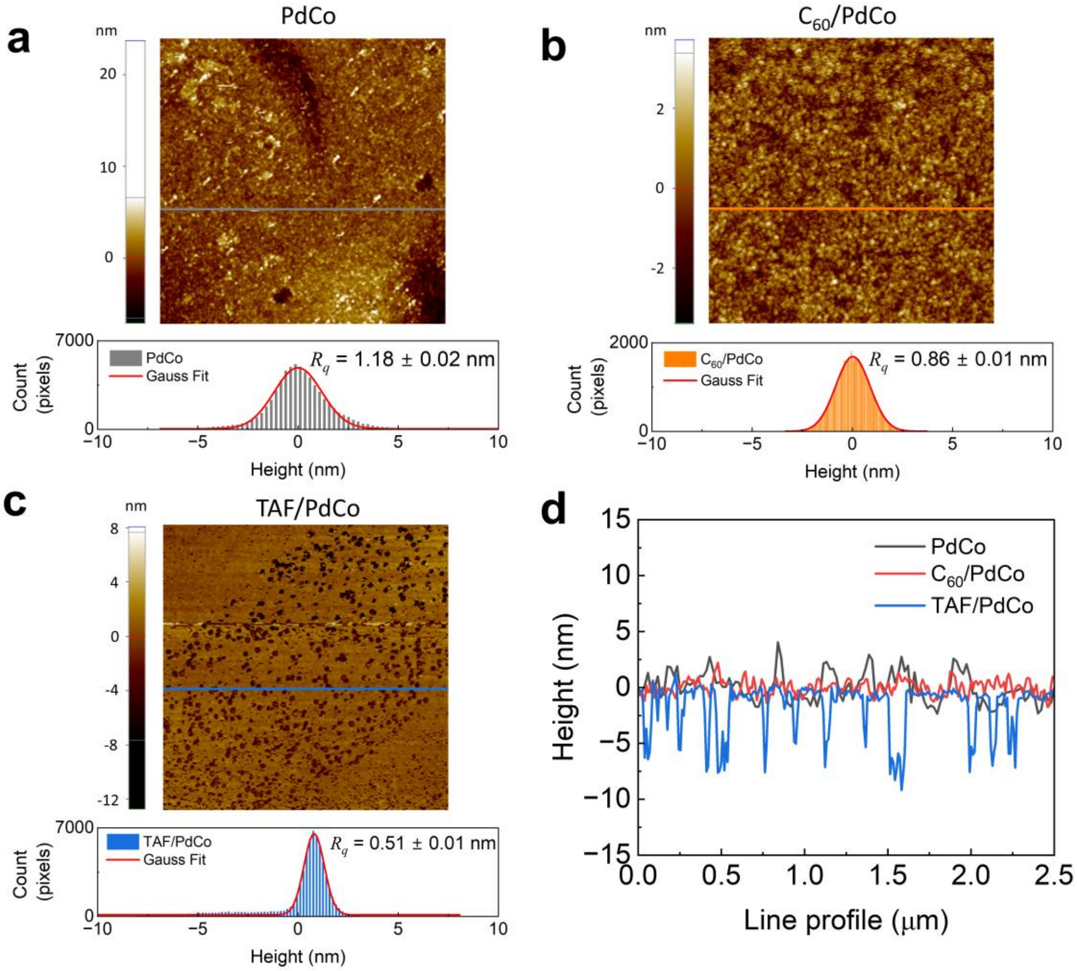

Supplementary Figure 9. AFM images of 5 nm PdCo thin film on **a** a glass substrate, **b** a 50-nm C_{60}-coated glass subtrate, **c** a 30-nm TAF-coated glass substrate and the corresponding histograms. **d** The line profiles across the films extracted from figures (a-c). All microscopy images were obtained in triplicate (N = 3) and display similar results. Source data are provided as a Source Data file.

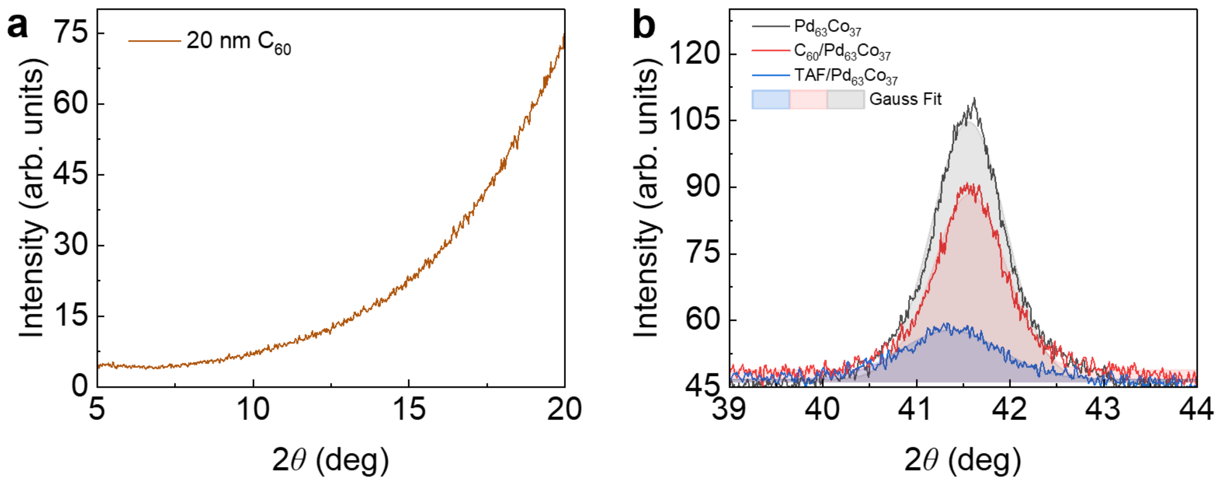

Supplementary Figure 10. XRD spectra of **a** 20 nm C_{60} thin film and **b** 15 nm $Pd_{63}Co_{37}$ thin films on different coated glass substrates. Source data are provided as a Source Data file.

Supplementary Table 4. Crystal structure and other physical properties of 15 nm $Pd_{63}Co_{37}$ thin films on different coated glass substrates extracted from XRD.

Sample	Peak position	FWHM	Crystallite size	Lattice Strain	d-spacing	Lattice constant
	2θ (deg)	β (deg)	D (nm)	ε (%)	d (Å)	a (Å)
**PdCo**	41.559 ± 0.002	0.93 ± 0.01	9.14 ± 0.06	1.07 ± 0.01	2.17 ± 0.01	3.76 ± 0.03
**C_{60}/PdCo**	41.571 ± 0.003	0.90 ± 0.01	9.55 ± 0.07	1.02 ± 0.01	2.17 ± 0.02	3.76 ± 0.03
**TAF/PdCo**	41.364 ± 0.007	1.25 ± 0.02	6.80 ± 0.10	1.45 ± 0.01	2.18 ± 0.03	3.78 ± 0.06

Thermal evaporated C_{60} thin film on non-epitaxial substrates like glass often leads to a mix of polycrystalline and amorphous-like regions.[46] Thus, no peak was observed in XRD spectrum of 20 nm C_{60} thin film in Supplementary Fig. 10a, confirming the absence of long-range crystalline order.[47] The C_{60} film is not perfectly close-packed but exhibits partial ordering with voids and grain boundaries. It is supported by Samad *et al*. where the porosity of C_{60} thin films was reported to be more than 50% when the substrate was kept at room temperature during the deposition.[48]

PdCo thin films deposited on different coated substrates exhibit the strongest Bragg diffraction peaks between 41.3° and 41.6°, which can be attributed to the (111) orientation of the face-centered cubic (fcc) PdCo lattice (Supplementary Fig. 10b). These peaks are shifted to higher angles relative to the (111) peak of pure fcc Pd (~40.2°), indicating that Co atoms are substitutionally incorporated into the Pd lattice. The observed peak positions and extracted lattice constants are consistent with prior literature report by Morgan *et al*.[49] Analysis of the full width at

half maximum (FWHM) values allowed for the estimation of crystallite size and lattice strain, as summarized in the accompanying Supplementary Table 4. Notably, the C_{60}/PdCo sample exhibits a larger crystallite size and reduced lattice strain compared to PdCo grown directly on glass, likely due to the smoother growth surface provided by the C_{60} layer. Although slightly larger grains typically correlate with slower sensor response, the C_{60}/PdCo sample demonstrated enhanced sensitivity and faster response times (see main text **Fig. 2**), suggesting that gas diffusion through the nanoporous C_{60} interlayer plays a critical role. In contrast, TAF/PdCo showed the lowest 2θ value, indicative of lattice expansion due to tensile strain, which can be attributed to pinholes in the TAF film, as observed in AFM images (Supplementary Fig. 9). The improved sensing response of samples with TAF underneath appears to result from a combination of reduced grain size, efficient gas permeation through the underlayer,[50] and a lowered energy barrier for hydrogen sorption at the TAF/PdCo interfaces[15].

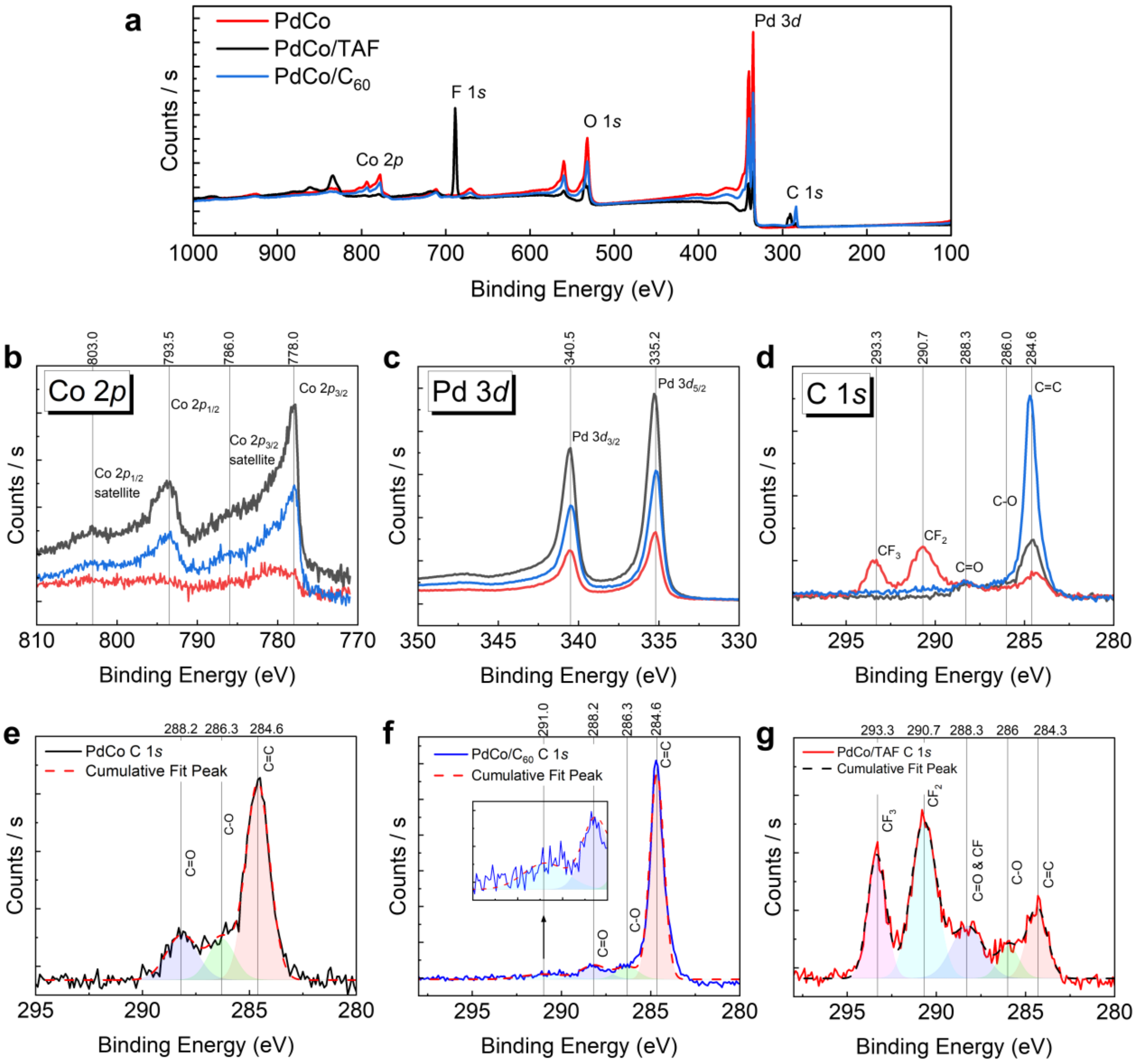

Supplementary Figure 11. X-ray photoelectron spectroscopy (XPS) analysis of PdCo thin films with and without molecular coatings. **a** Full survey spectra for 15 nm PdCo (black), PdCo/2 nm C_{60} (blue), and PdCo/2 nm TAF (red). **b–d** High-resolution spectra of Co 2*p*, Pd 3*d*, and C 1*s* regions respectively, highlighting the changes in chemical states and bonding environments upon C_{60} and TAF coating. **e–g** Deconvoluted C 1*s* spectra for bare PdCo, PdCo/C_{60}, and PdCo/TAF, respectively. Source data are provided as a Source Data file.

To investigate the surface modification and gain deeper insight into chemical bonding and electronic interactions between the PdCo layer and molecular coatings, we conducted X-ray photoelectron spectroscopy (XPS) analysis of PdCo thin films with and without surface functionalization, as shown in Supplementary Figure 11. The full survey spectra (Supplementary Fig. 11a) reveal characteristic peaks corresponding to Pd 3*d*, Co 2*p*, C 1*s*, O 1*s*, and F 1*s*. A comparative analysis of Co 2*p* (Supplementary Fig. 11b) and Pd 3*d* (Supplementary Fig. 11c) regions between the bare PdCo and PdCo/C_{60} samples shows negligible differences. No peak shifts or changes in the peak-to-peak intensity ratio were observed, indicating minimal impact on the Pd and Co core levels upon C_{60} deposition. In the PdCo/TAF spectrum, the Co 2*p* and Pd 3*d* intensities are markedly reduced, consistent with attenuation effects arising from the overlying TAF layer, which reduces both incident X-ray penetration and photoelectron emission due to its insulating character.

The C 1s region, however, displays the most pronounced differences among the samples (Supplementary Fig. 11d). Deconvoluted C 1*s* spectra for PdCo, PdCo/C_{60}, and PdCo/TAF are shown in Supplementary Fig. 11e-g, respectively. All samples were exposed to ambient laboratory conditions for several months; thus, the C 1*s* signal in bare PdCo shows typical adventitious carbon contamination, including peaks corresponding to C–C (~284.6 eV), C–O (~286.3 eV), and C=O (~288.2 eV), likely due to atmospheric CO_2 or CO adsorption.[51]

Upon deposition of a 2 nm C_{60} layer, the C 1s intensity increases significantly, and the dominant peak at ~284.6 eV remains, consistent with the sp^2-hybridized carbon bonds of C_{60} molecules.[52] Additional weak features in the 288–292 eV range (Supplementary Fig. 11f) suggest the emergence of interfacial bonding environments such as Pd–C or Co–C.[52] Combining these insights with the results presented in Supplementary Fig. 13, we note that although bonding occurs at the C_{60}/PdCo interface, the catalytic active Pd sites for H_2 adsorption are not fully passivated. As a result, the sensor with the C_{60} top layer exhibits comparable sensing performance to the uncoated counterpart. This indicates that C_{60} does not significantly alter the activation energy for H_2 adsorption. Instead, the fast diffusion of H_2 molecules through the porous C_{60} matrix appears to be the dominant factor contributing to the preserved sensing kinetics.

In the case of TAF-functionalized PdCo (Supplementary Fig. 11g), the PdCo/TAF spectrum exhibits a distinct F 1*s* peak at ~688 eV and its C 1*s* spectrum displays complex features spanning 284 – 293 eV. In addition to the typical C–C and C=O peaks, strong peaks at ~290.7 eV and ~293.3 eV correspond to CF_2 and CF_3 moieties, respectively, confirming the formation Pd-CF_x bonds.[53] As a result, TAF coating modifies the Pd surface chemistry, lowering the activation energy for H_2 adsorption and thereby enhancing sensor responsiveness.[15]

Supplementary Note 6. Sensing characteristics of CHAs on different C_{60} thicknesses

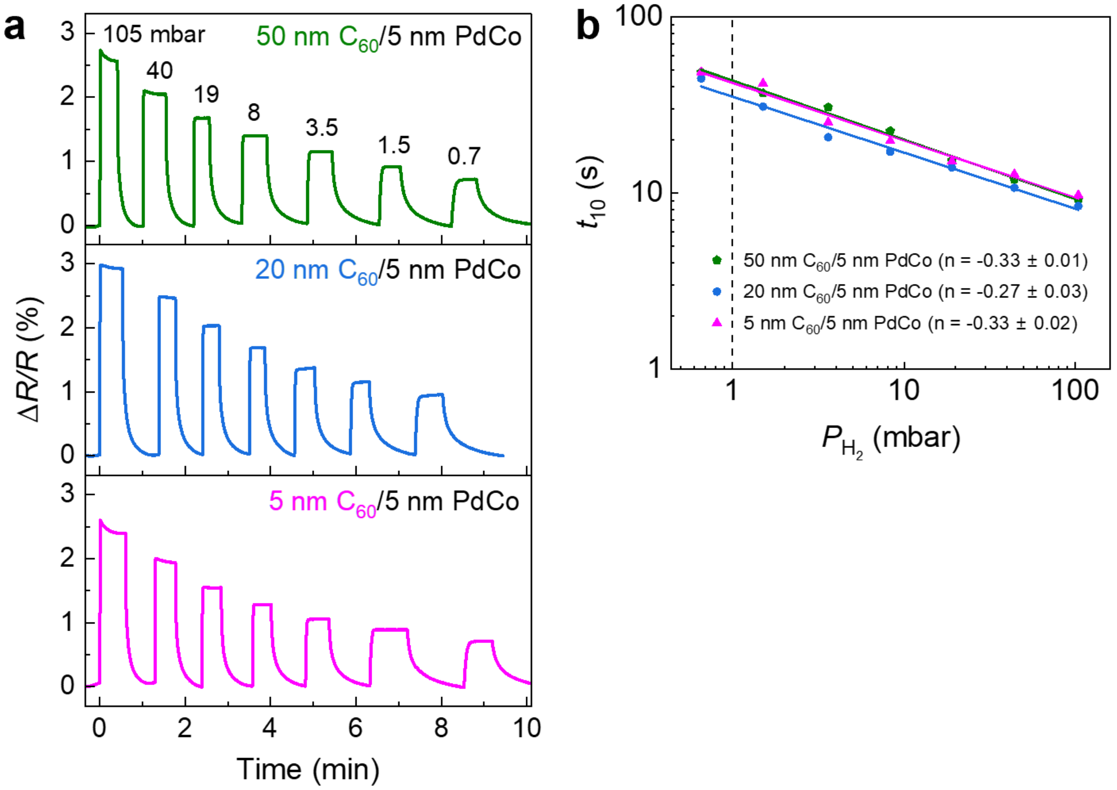

Supplementary Figure 12. Sensing performances of C_{60}/5 nm PdCo CHA_{450} sensors with different C_{60} thicknesses. a Sorption dynamics in response to step wise decreasing H_2 pressure from 105 to 0.7 mbar. **b** Release time t_{10} extracted from (a). All measurements were performed in vacuum mode at room temperature. Source data are provided as a Source Data file.

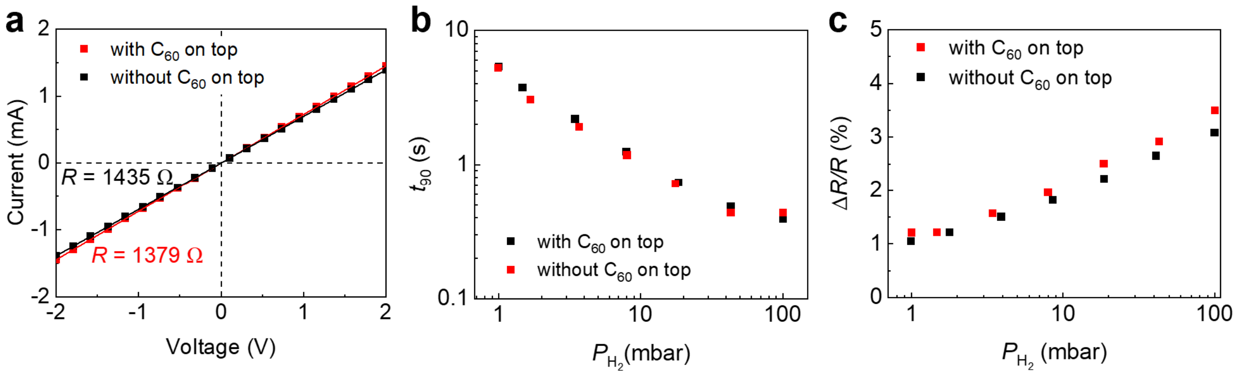

Supplementary Figure 13. Sensing performances of 20 nm C_{60}/5 nm PdCo CHA_{300} sensors with and without 20 nm C_{60} on top. a *I-V* characteristics, **b** response time t_{90} and **c** sensitivity of the sensors in response to step wise decreasing H_2 pressure from 100 to 1 mbar. All measurements were performed in vacuum mode at room temperature. Source data are provided as a Source Data file.

Supplementary Note 7. CHAs with different etching time t_{RIE}

Supplementary Note 7.1. Morphology characterization and glancing angle deposition (GLACD) simulation

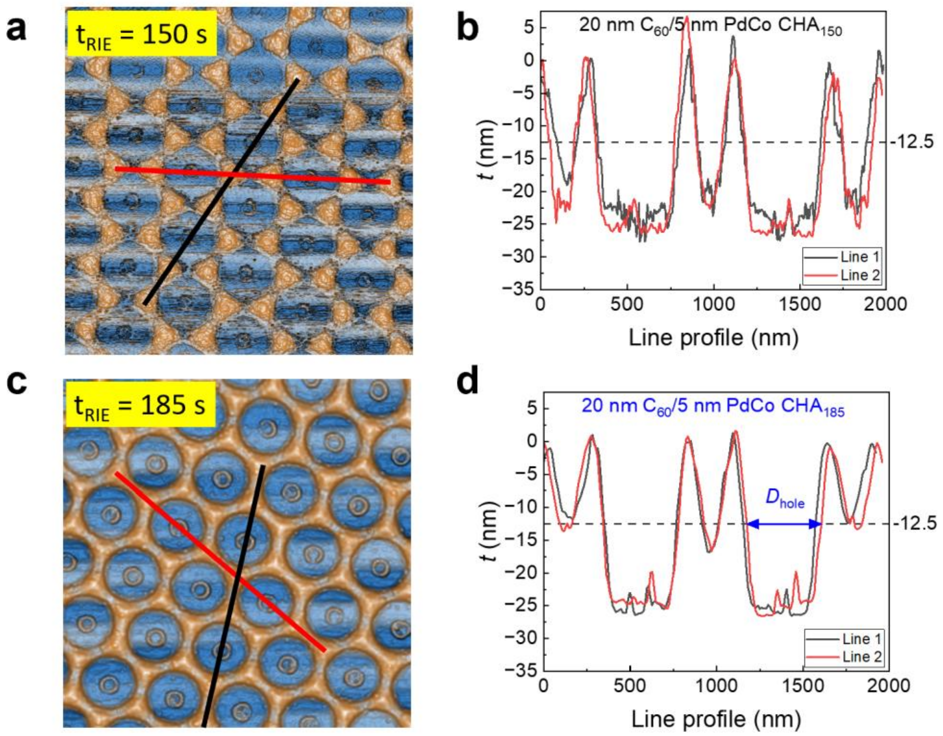

Supplementary Figure 14. **a&c** Top-view AFM image of CHA_{150} and CHA_{185} and **b&d** the corresponding line profile along 2 lines denoted in (a)(c). The hole diameter D_{hole} is extracted from the middle of the hole and is averaged from 4 holes as depicted in (d). All microscopy images were obtained in triplicate (N = 3) and display similar results. Source data are provided as a Source Data file.

When etching time < 160 s (Supplementary Fig. 14 a-b), a nanotriangle array was achieved instead of a nanohole array due to the pronounced shadow effect from the big PS beads, resulting in a discontinuous nano network. The hole diameters of all CHAs were extracted from AFM line profiles (similar to CHA_{185} depicted in Supplementary Fig. 14 c-d) and summarized in Supplementary Table 5. D_{hole} was then used as input parameters for GLACD simulation.[54]

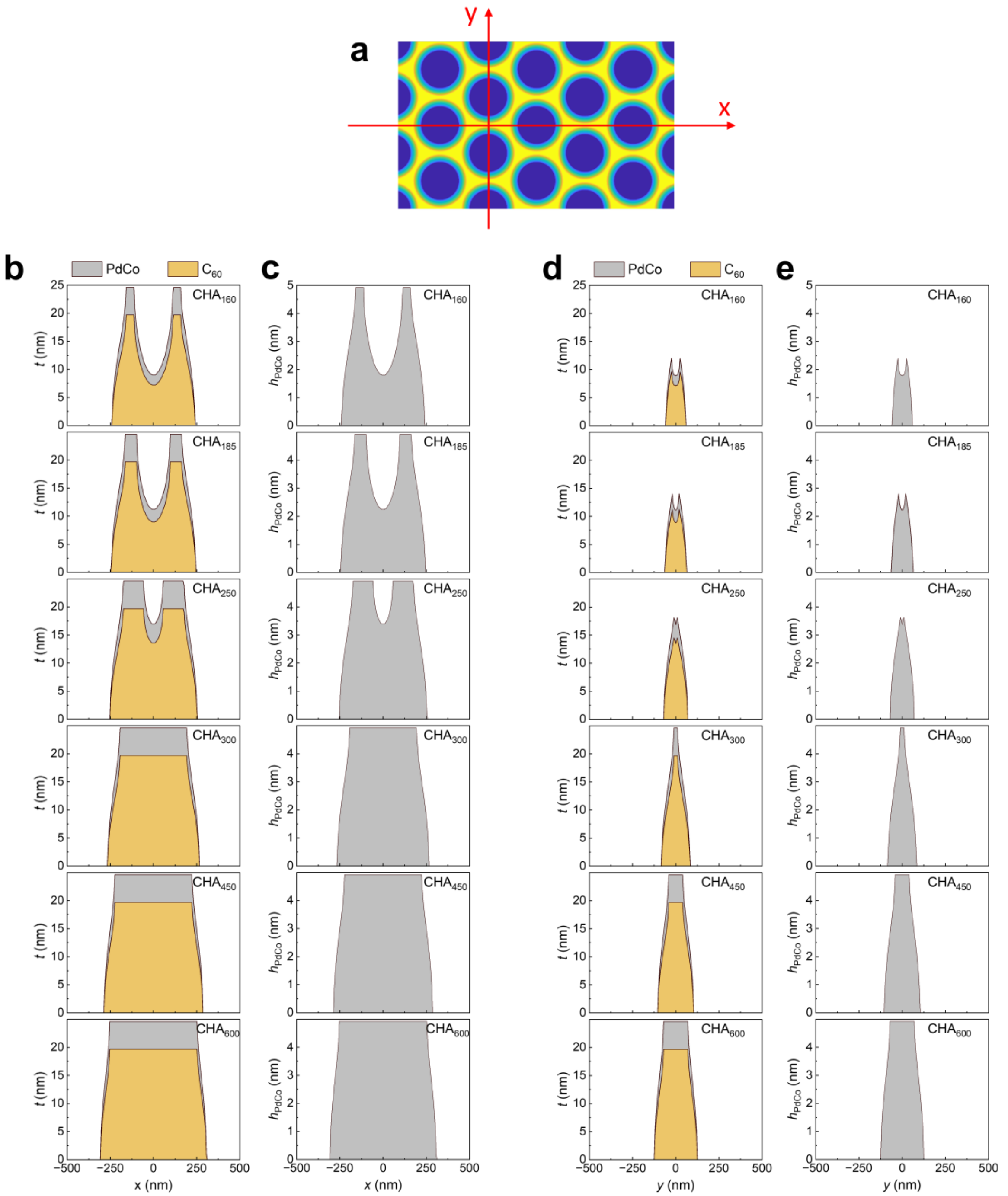

Supplementary Figure 15. **a** Simulated hole array diagram and the bottleneck cross-section for each $CHA_{t_{RIE}}$ along **b** the x-axis and **d** the y-axis denoted in (a). **c** and **e** are the thicknesses of the PdCo layer in (b) and (d) projected on a flat substrate. Source data are provided as a Source Data file.

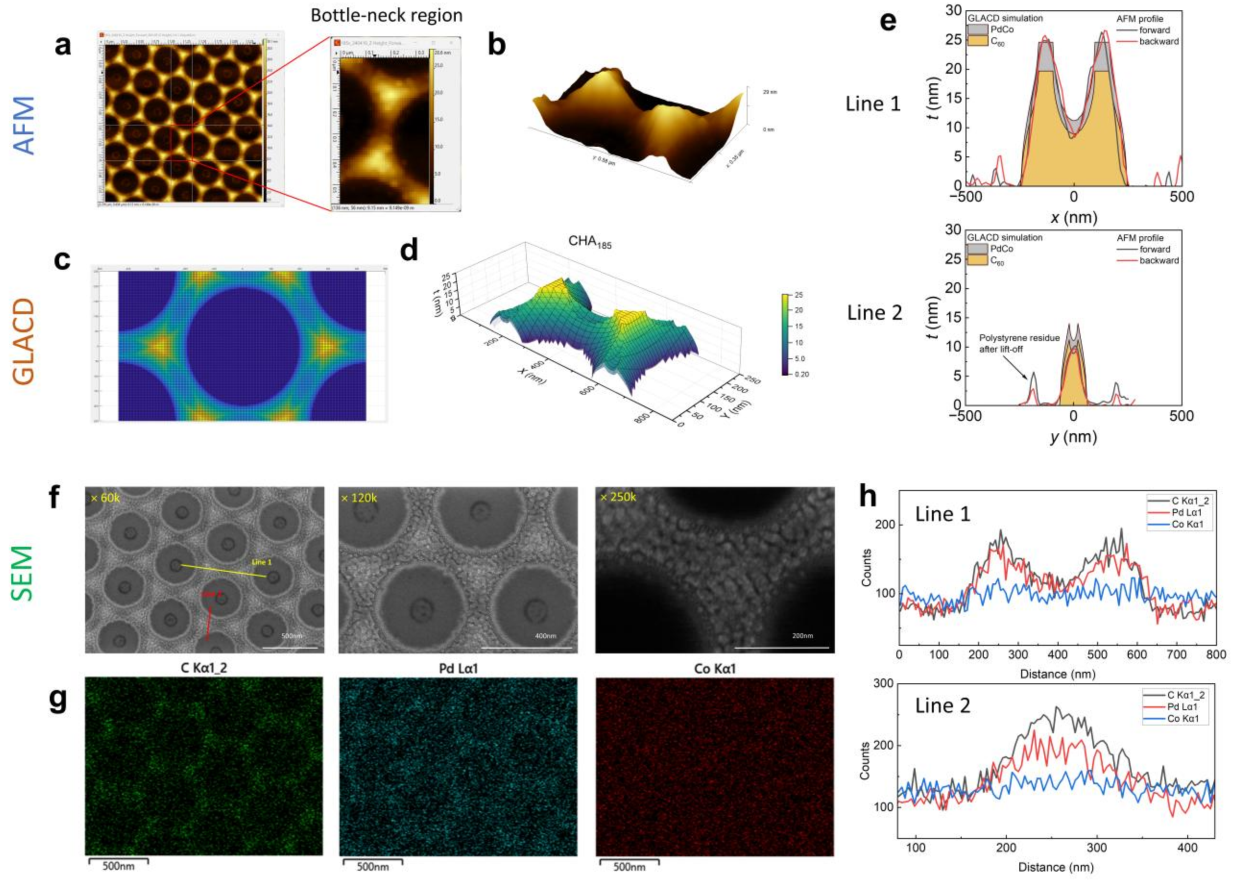

Supplementary Figure 16. Comparison between GLACD simulation and data from AFM and SEM. **a** Top-view and **b** side-view AFM images of CHA_{185}. **c** Top-view and **d** side-view of CHA_{185} from GLACD simulation. **e** Line profiles along (line 1) and across (line 2) the bottle-neck region. **f** SEM images of 20 nm C_{60}/5 nm PdCo CHA_{185} at different magnifications. The estimated diameter of PdCo grains on the surface is $\gamma_{particle} = 25 \pm 1$ mm. **g** Energy-dispersive spectroscopy (EDS) elemental mapping. **h** EDS line spectra along (line 1) and across (line 2) the bottle-neck region denoted in (f). Counts are based on weight percentages. All microscopy images were obtained in triplicate (N = 3) and display similar results. Source data are provided as a Source Data file.

Supplementary Note 7.2. Surface-to-volume (SVR) ratio calculation

The morphology of each layer in CHAs was simulated using an in-house glancing angle deposition simulation.[54] The result of the simulation on the substrate for one unit cell is depicted in Supplementary Fig. 16c with a resolution of 173 x 100 pixels^2. For polystyrene (PS) monolayer with a bead diameter of 500 nm, each pixel has a size of 5 x 5 nm^2. The color bar represents the thickness t of the deposited materials. The morphology of each layer can be illustrated as a 3D surface (Supplementary Fig. 16d), and its surface area is calculated using *surfacearea* function in MATLAB.[55] The total surface area of PdCo layer can be calculated as

$$\text{Total PdCo surface} = \text{Top surface area of PdCo} + \text{Top surface area of } C_{60}. \quad \text{(S2)}$$

The volume of each layer is $V_{\text{layer}} = 5 \times 5 \times t$ (nm^3), thus the volume of PdCo layer is

$$V_{\text{PdCo}} = V_{\text{total}} - V_{\text{C60}}, \quad \text{(S3)}$$

where V_{total} is total volume of the device C_{60}/PdCo and V_{C60} is volume of the C_{60} layer only.

Finally, the surface-to-volume ratio is calculated as

$$\text{SVR} = \text{Total PdCo surface}/V_{\text{PdCo}}. \quad \text{(S4)}$$

Supplementary Table 5. Summary of all parameters of 20 nm C_{60}/5 nm PdCo $CHA_{t_{RIE}}$ extracted from AFM images and GLACD simulations.

Etching time	Hole diameter	Bottle-neck width	Bottle-neck thickness of PdCo	Cross-sectional area	Bottle-neck length	Surface to volume ratio	Resistance	
t_{RIE}	D_{hole}	w	h_{PdCo}	$A_{bottleneck}$	l	SVR	$R_{without\ TAF}$	$R_{with\ TAF}$
s	nm	nm	nm	nm^2	nm	nm^{-1}	Ω	Ω
160	460 ± 3	40	2.00	1463.96	220	0.82	7062300	1889203
185	450 ± 4	50	2.46	1648.88	201	0.75	17744	15003
250	430 ± 8	70	3.55	2011.55	160	0.66	2835	2430
300	404 ± 2	96	4.92	2294.89	392	0.58	1503	1157
450	350 ± 5	150	4.92	2540.55	450	0.50	1093	1350
600	297 ± 6	203	4.92	2786.28	512	0.46	435	637

Supplementary Note 7.3. Resistivity model

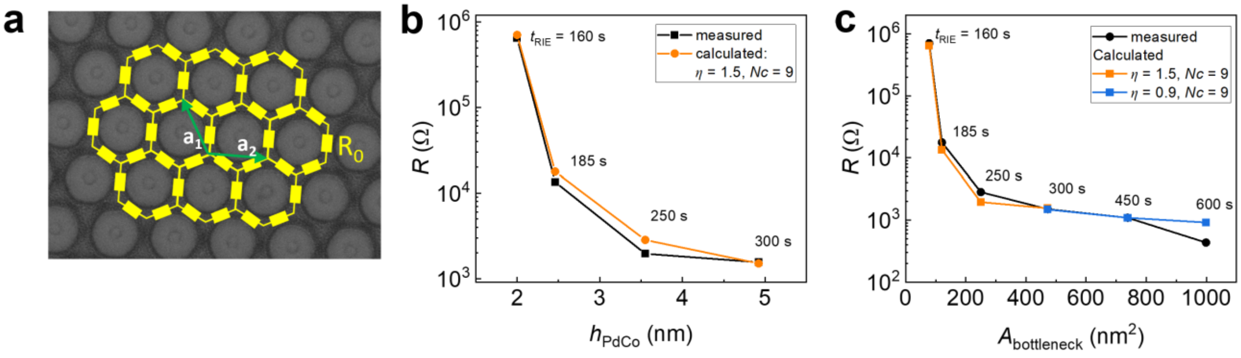

Supplementary Figure 17. **Resistivity model for 20 nm C_{60}/5 nm PdCo $CHA_{t_{RIE}}$.** **a** Schematic of the infinite two-dimensional hexagonal lattice of identical resistors R_0. **b-c** Comparison between thickness-dependent resistance and Lacy's model (Ref. 57).

A hexagonal CHA structure can be considered as an infinite honeycomb resistor network of identical unit resistors (Supplementary Fig. 17a); and its resistance R_{NH} is directly proportional to the elementary resistance R_0:

$$R_{\mathrm{NH}} = NR_0, \quad \text{(S5)}$$

where the constant N is determined by

$$N = \frac{3}{4\pi^3}\int_{-\pi}^{\pi}\int_{-\pi}^{\pi}\frac{1-\cos(mx+ny)}{3-\cos(x)-\cos(y)-\cos(x+y)}dxdy\,. \quad \text{(S6)}$$

R_{NH}, here, is calculated spaced; the origin (0,0) and a given lattice point (m,n) of a two-dimensional hexagonal resistor network.[56] Since the clip test's pins using for 4-point-probe measurements are equally spaced, N should be a constant and has the same value for all devices. The effective resistance of the elementary resistor can be estimated by the following equation:

$$R_0 = \rho\frac{l}{w.h_{PdCo}} \quad \text{(S7)}$$

with ρ is the resistivity of PdCo alloy; l, w, and h_{PdCo} are the length, width, and thickness of PdCo layer (Supplementary Table 5). Note that the value of the elementary resistance is determined solely by the intersection of the narrowest (w) and thinnest bottleneck region (h_{PdCo}).[13]

When the film thickness h_{PdCo} is smaller than the electronic mean free path in bulk l_{bulk}, the resistivity ρ is no longer a constant equal to the bulk resistivity ρ_0, but increases nonlinearly with the decrease of film's thickness. Lacy[57] had developed a general model that demonstrates the dependent of ρ on the film thickness, the surface roughness, and the grain boundary of the metallic thin film:

$$\rho = \frac{c\rho_0}{\kappa\prime(1-\ln(\kappa'))}, \qquad \text{(S8)}$$

in which

$$\kappa' = \frac{t_{PdCo}-\eta}{2l_{bulk}}. \qquad \text{(S9)}$$

In this model, c is a correction factor for the scattering from impurities in films ($c > 1$), and η is a thickness correction factor that accounts for the scattering from the surfaces, grain boundaries and surface roughness ($\eta < t_{PdCo}$). Thus, R_{NH} can be calculated using Lacy's model by substituting equations (S7 – S9) into (S5):

$$(R_{NH})_{calculated} = \frac{Nc}{\frac{t_{PdCo}-\eta}{2l_{bulk}}\left(1-\frac{t_{PdCo}-\eta}{2l_{bulk}}\right)}\rho_0\frac{l}{wt_{PdCo}}. \qquad \text{(S10)}$$

Using the values in Table S2 with fixed $l_{bulk} = 20$ nm and $\rho_0 = 6.72 \times 10^{-8}$ (Ωm), the thickness-dependent resistivities of $CHA_{t_{RIE}}$ (t_{RIE} = 160 s to 300 s) are shown in Supplementary Fig. 17b. The theoretical model best fits experimental data when η = 1.5 nm and Nc = 9.0 for this regime. The small mismatch here could be explained by (i) the complexity of the CHA structure with non-uniformed film thickness and (ii) the assumption that the contribution of the triangle regions is negligible compared to the bottle-neck regions. For t_{RIE} > 300 s, t_{PdCo} remains unchanged, therefore R_{NH} was plotted versus the cross-sectional area of the bottleneck region in Supplementary Fig. 17c with η = 0.9 nm and Nc = 9.0. Here, a smaller fitting parameter η than the ultra-thin film regime was used indicating that the scattering effects at the surface/grain

boundaries/impurities is less serious in thicker films. Overall, the non-linear dependency of CHAs' electrical resistance on the thickness or the bottleneck size being observed in the experiment was explained through Lacy's resistivity model.

Supplementary Note 7.4. Sensing characteristics of CHAs with different etching time t_{RIE}

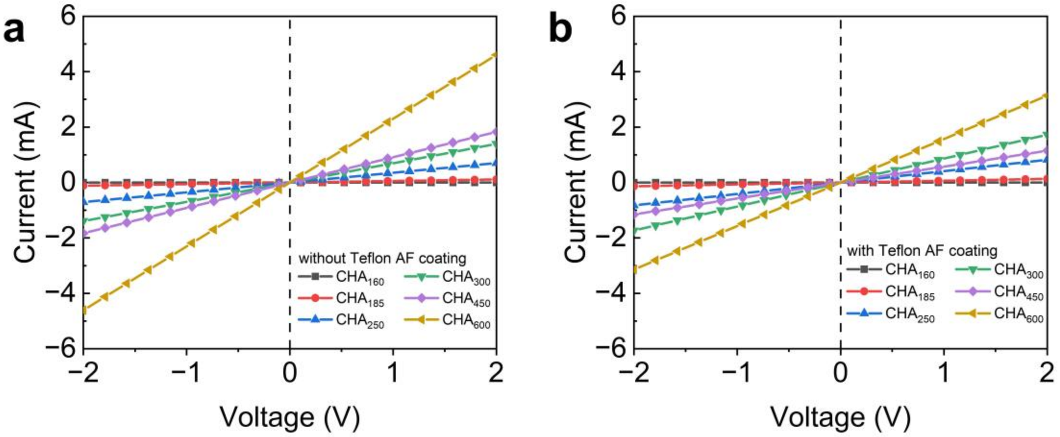

Supplementary Figure 18. *I-V* characteristics of $\mathrm{CHA}_{t_{\mathrm{RIE}}}$ **a** without and **b** with TAF coating. Source data are provided as a Source Data file.

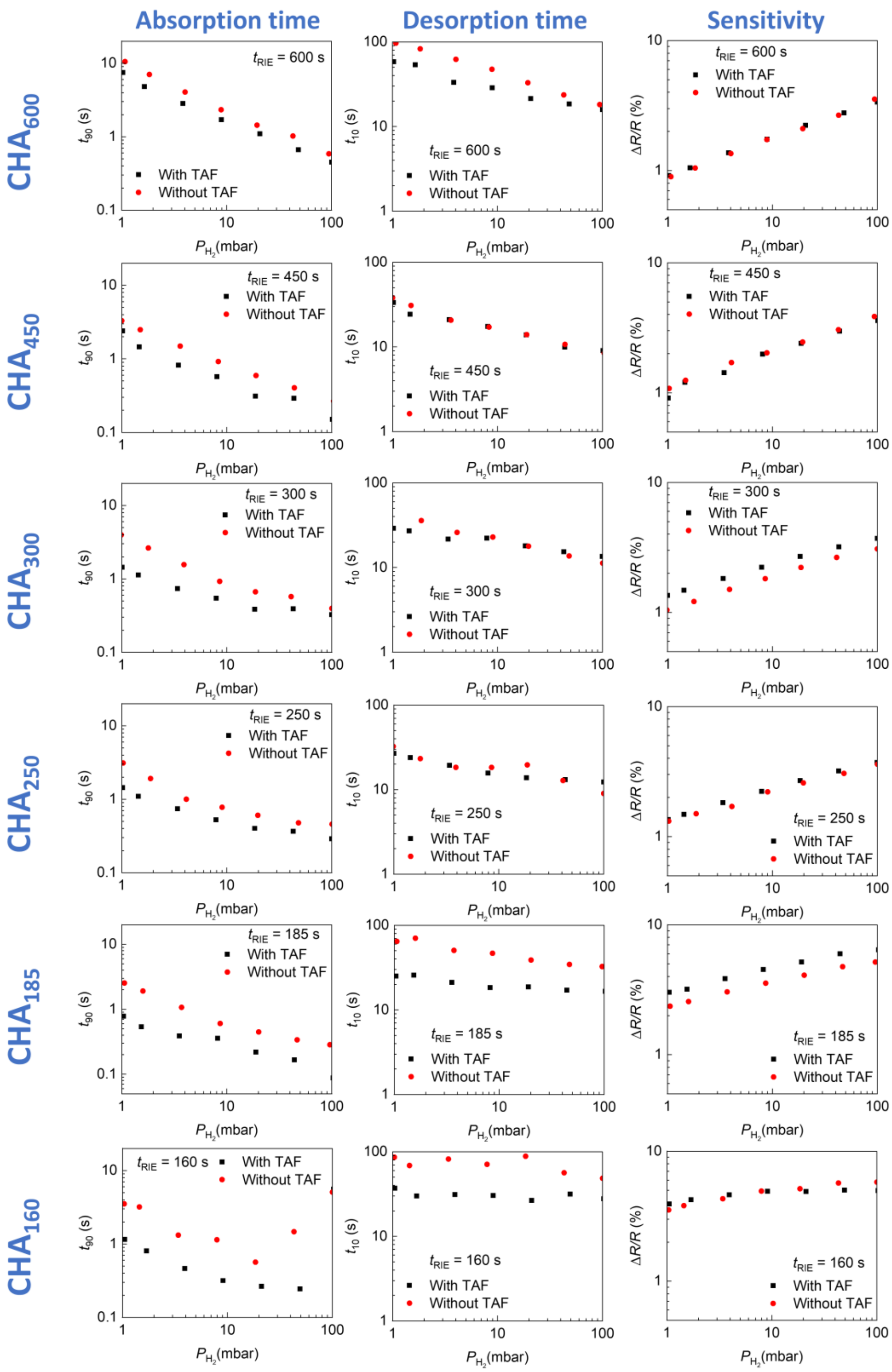

Supplementary Figure 19. Absorption times, desorption times and sensitivities of 20 nm C_{60}/5 nm PdCo/(30 nm TAF) $CHA_{t_{RIE}}$ measured in vacuum mode at room temperature. Source data are provided as a Source Data file.

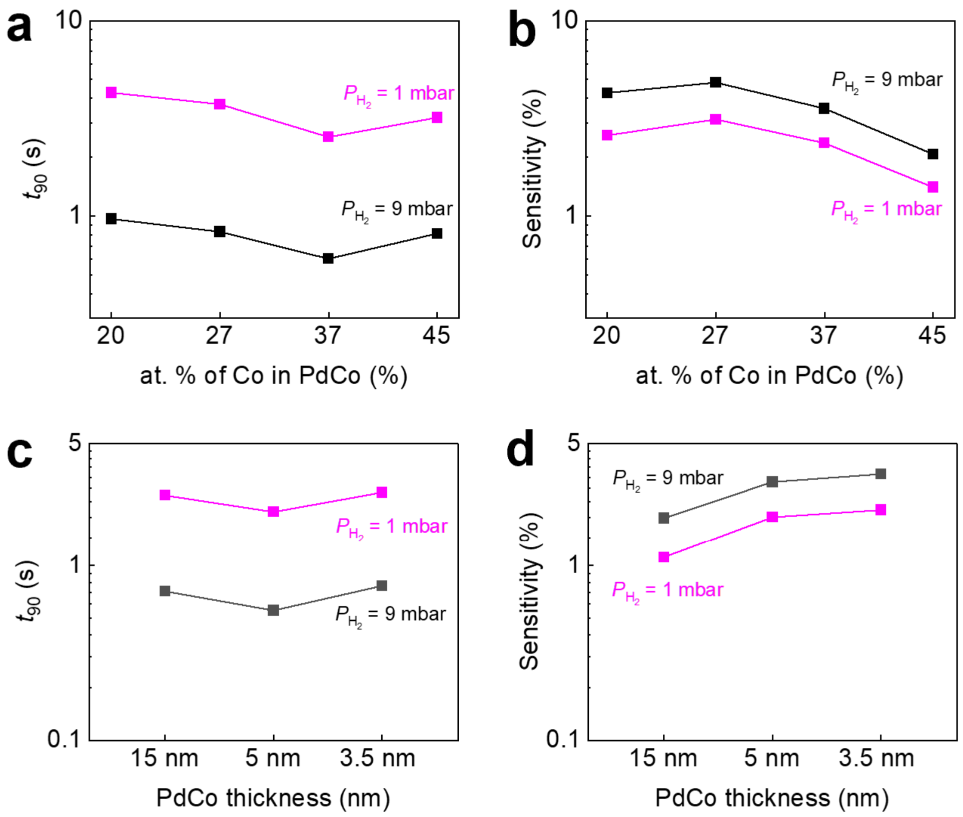

Supplementary Figure 20. **a-b** Pd:Co composition-dependent and **c-d** PdCo thickness-dependent sensing performances of 20 nm C_{60}/PdCo CHA_{185}. Source data are provided as a Source Data file.

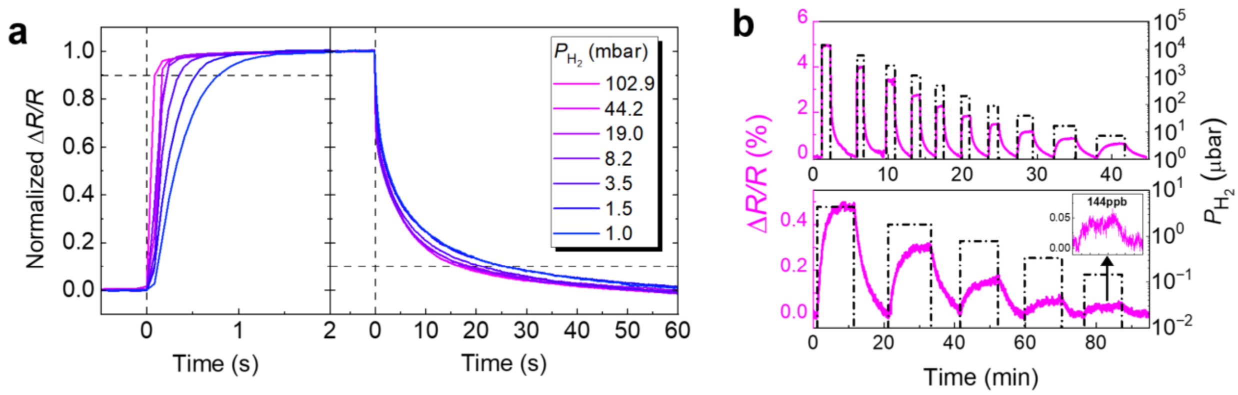

Supplementary Figure 21. Sensing performances of 20 nm C_{60}/5 nm PdCo/30 nm TAF CHA_{185}. a Normalized absorption/desorption (left/right) kinetic of the sensor in response to varying H_2 pressure from 100 to 1 mbar measured at 12.2 Hz sampling frequency. **b** Δ*R/R* response to stepwise decreasing partial H_2 pressures of (top) 13880 – 7.2 μbar and (bottom) 4.32 – 0.144 μbar measured at 8.4 Hz sampling frequency. All measurements were performed in vacuum mode at room temperature. Source data are provided as a Source Data file.

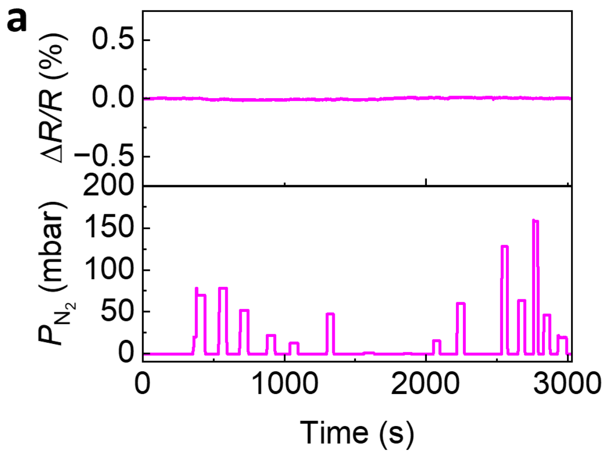

Supplementary Figure 22. Controlled experiments of **a** 20 nm C_{60}/5 nm PdCo/30 nm TAF CHA_{185} sensor and **b** 20 nm C_{60}/3nm TAF/5 nm PdCo/30 nm TAF CHA_{450} sensor with pure N_2. Top panel shows the sensor's response to the corresponding step-wise N_2 pressure pulses (from 0.1 to < 200 mbar) in the bottom panel. The experiment were performed in vacuum mode at room temperature and at sampling frequency $f_{sampling}$ = 12.2 Hz. Source data are provided as a Source Data file.

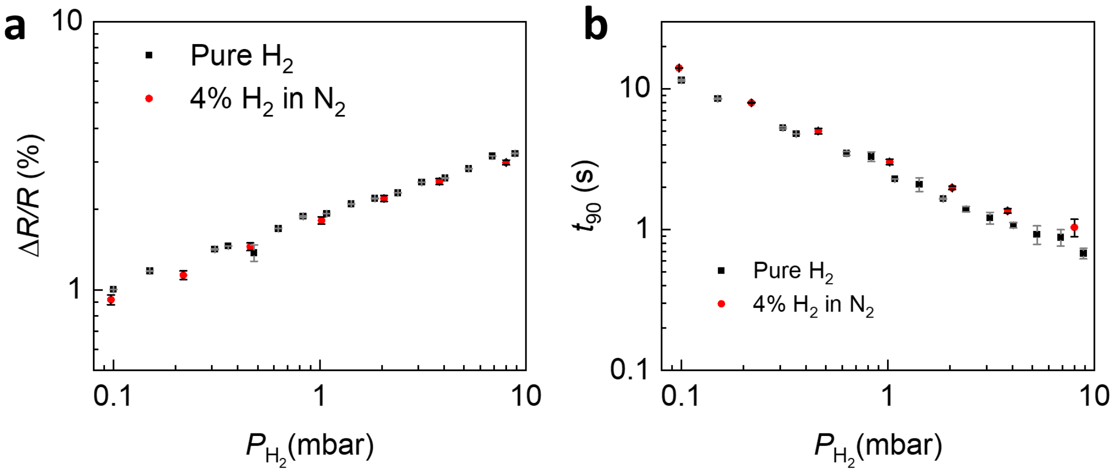

Supplementary Figure 23. **a** Sensitivity $\Delta R/R$ and **b** absorption time t_{90} of of 20 nm C_{60}/5 nm PdCo CHA_{300} sensor measured in vacuum mode using pure H_2 and 4% H_2 in N_2 balance as the gas sources. Data are presented as mean values +/- SEM. Source data are provided as a Source Data file.

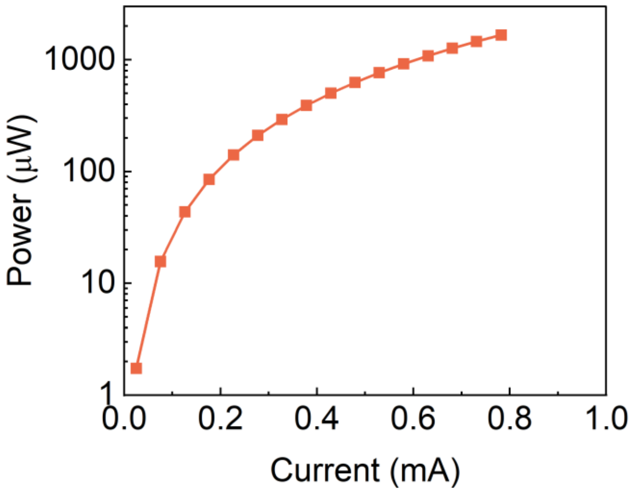

Supplementary Figure 24. Measured power of the 20 nm C_{60}/3nm TAF/5 nm PdCo/30 nm TAF CHA_{450} sensor. Source data are provided as a Source Data file.

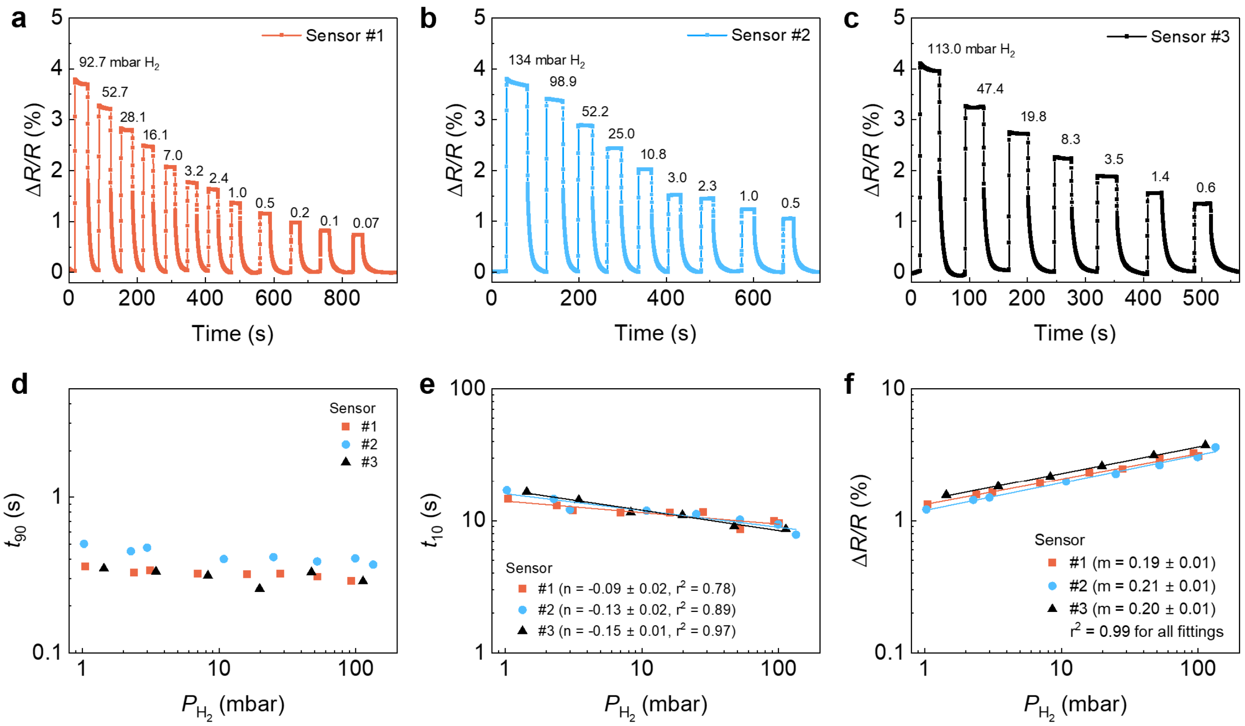

Supplementary Figure 25. Sensing performances of 20 nm C_{60}/3 nm TAF/5 nm PdCo/30 nm TAF CHA_{450} sensors. a-c $\Delta R/R$ responses of 3 sensors with the same device structure to stepwise decreasing H_2 pressures of ~100 to < 1 mbar ($f_{sampling}$ = 12.2 Hz). **d** Absorption time (t_{90}), **e** desorption time (t_{10}) and **f** sensitivity of the 3 sensors extracted from (a-c). All measurements were performed in vacuum mode at room temperature. Note that the data presented in Supplementary Fig. 24 d&e are identical with the ones shown in the main text Fig. 6b. Source data are provided as a Source Data file.

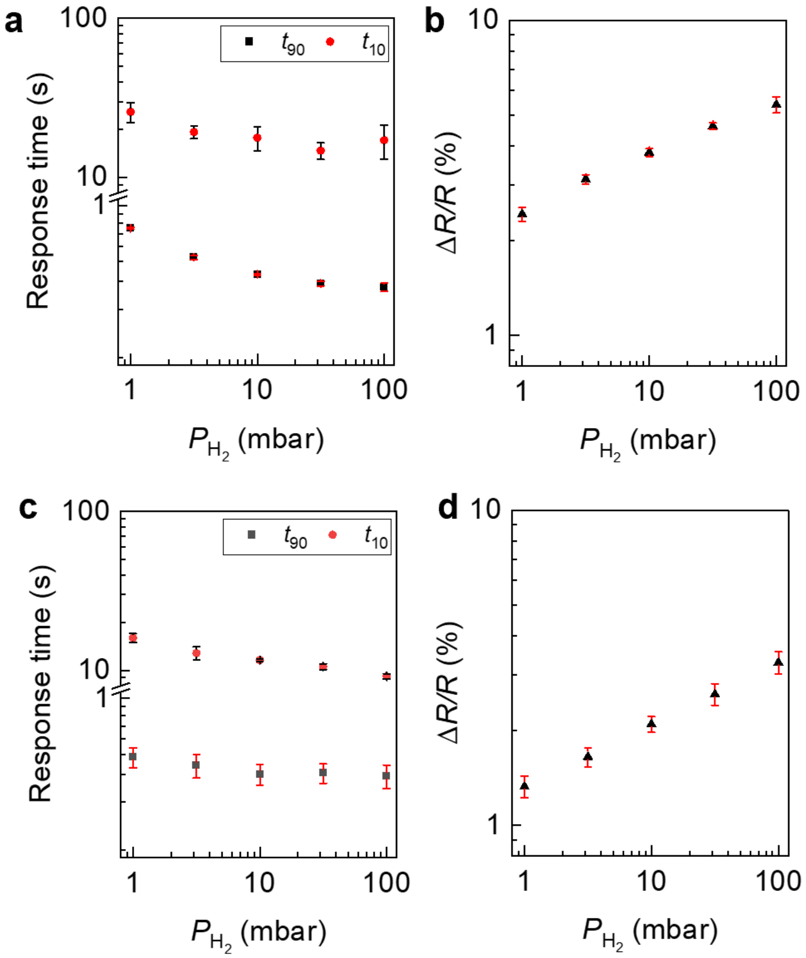

Supplementary Figure 26. **a** Average response time and **b** sensitivity of 20 nm C_{60}/5 nm PdCo/TAF CHA_{185} sensors. **c** Average response time and **d** sensitivity of 20 nm C_{60}/3 nm TAF/5 nm PdCo/TAF CHA_{450} sensors. The error bars indicate the standard deviation from 3 sensors. Data are presented as mean values +/- SD. Source data are provided as a Source Data file.

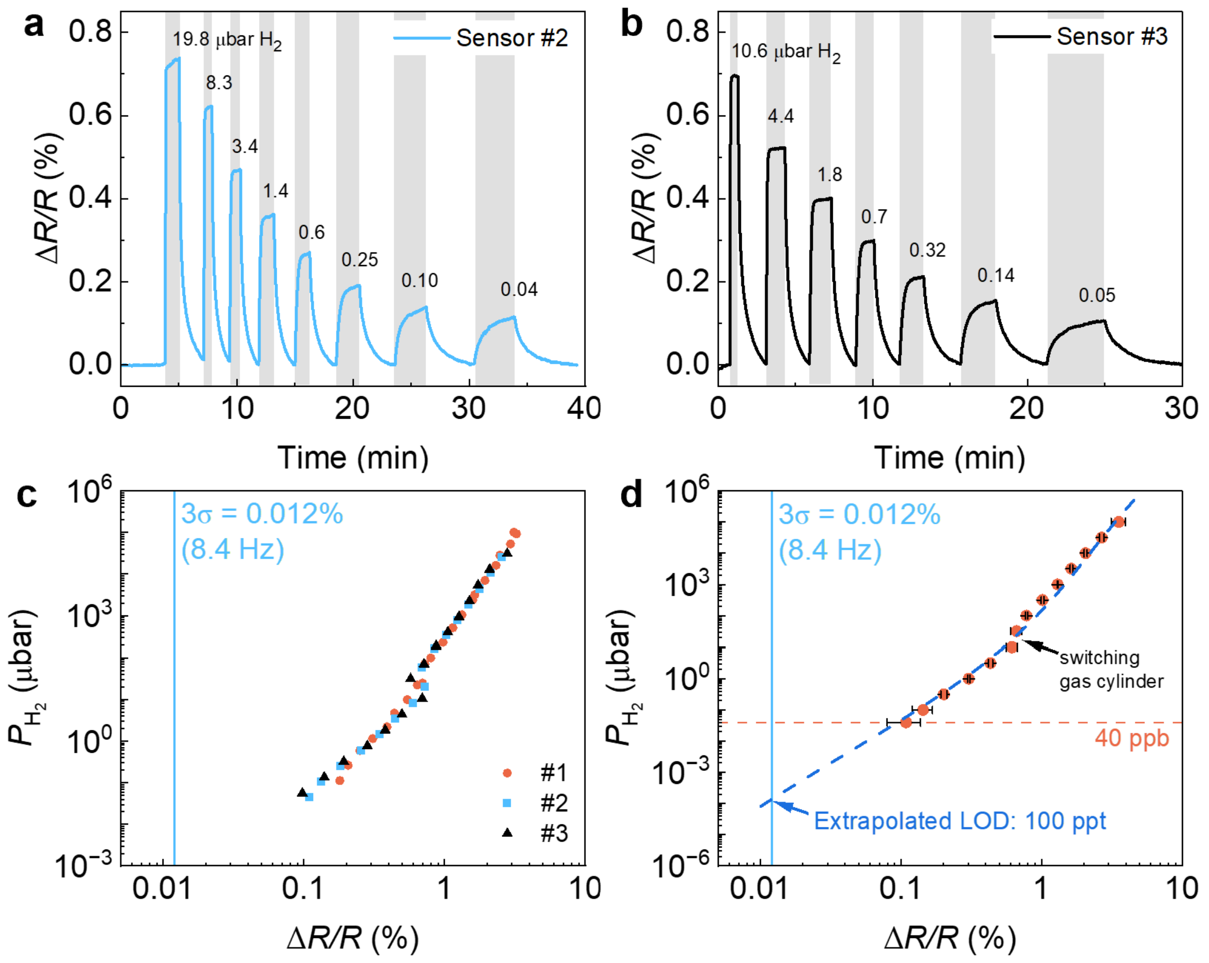

Supplementary Figure 27. LOD measurements of 20 nm C_{60}/3 nm TAF/5 nm PdCo/30 nm TAF CHA_{450} sensor. a, b Sensors' response at low H_2 pressure regime. **c** Measured sensitivities across 6 orders of H_2 pressure. **d** Averaged Δ*R/R* plot as a function of P_{H_2} with the error bars indicate the standard deviation from 3 devices. Data are presented as mean values +/- SD. The vertical blue line denotes the defined LOD of 3σ = 0.012% at 8.4 Hz sampling frequency (Supplementary Note 8). All measurements were performed in vacuum mode at room temperature. Source data are provided as a Source Data file.

Supplementary Note 8. Noise evaluation

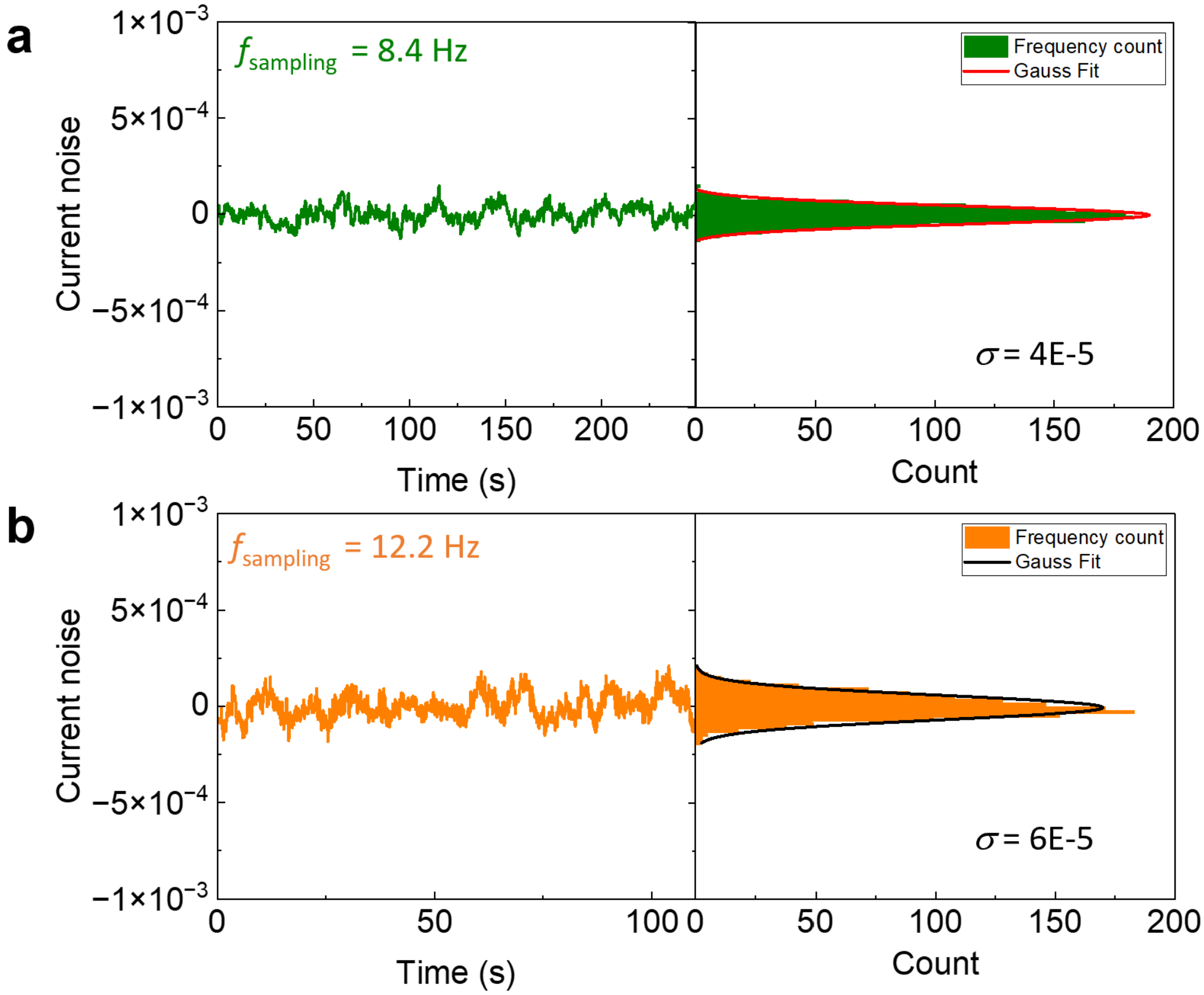

Supplementary Figure 28. (left panel) Experimental current noise or resistance noise (defined as ΔI/I = ΔR/R) versus acquisition time of the 20 nm C_{60}/5 nm PdCo/30 nm TAF CHA_{185s} at different sampling frequency: **a** $f_{sampling}$ = 8.4 Hz and **b** $f_{sampling}$ = 12.2 Hz. Δ*I* is the fluctuation of the electric current around the equilibrium current, *I*. **(right panel)** Histogram plot of signal intensity. By definition, the LOD is 3σ with σ is the standard deviation extracted from the Gaussian Fits. Thus, at $f_{sampling}$ = 8.4 Hz, 3σ = 0.012%; and at $f_{sampling}$ = 12.2 Hz, 3σ = 0.018%. Source data are provided as a Source Data file.

Supplementary Note 9. Stability, interference gases and humidity tests

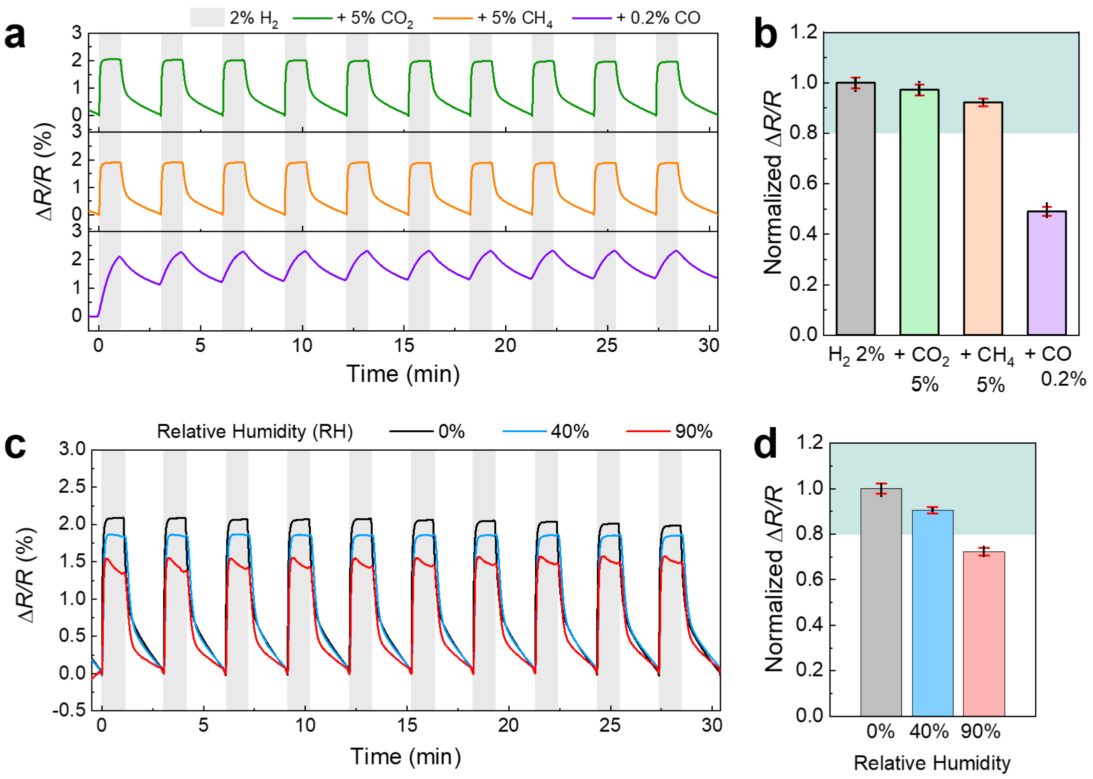

Supplementary Figure 29. Interference tests of 20 nm C_{60}/3 nm TAF/5 nm PdCo/TAF CHA450 (no PMMA coating) with different **a** gases and **c** relative humidity (RH) levels. **b** Normalized sensor signal to the one obtained with 2% H_2 in N_2 flow. **d** Normalized sensor signal to the one obtained with 2% H_2 in dry condition RH = 0%. The error bars in (b) and (d) indicate the standard deviation from 10 pulses. Data are presented as mean values +/- SD. All measurements were performed at room temperature in flow mode at a constant flow rate of 400 sccm and with N_2 as gas carrier. Source data are provided as a Source Data file.

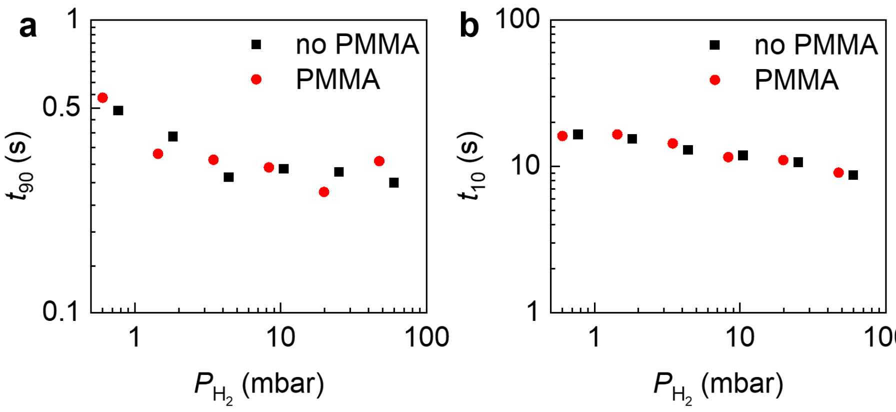

Supplementary Figure 30. a Response and **b** release time of 20 nm C_{60}/3 nm TAF/5 nm PdCo/TAF CHA450 with and without PMMA coating. Source data are provided as a Source Data file.

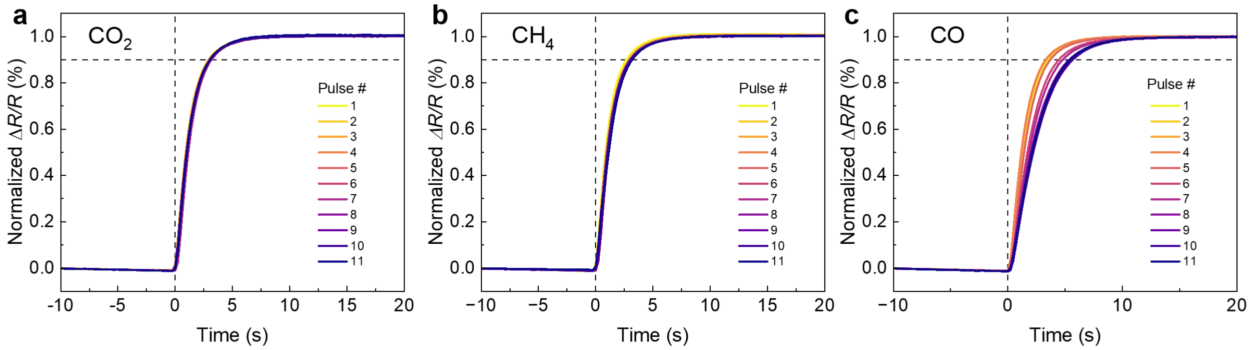

Supplementary Figure 31. Normalized resistance dynamics of 20 nm C_{60}/5 nm PdCo/TAF/PMMA CHA_{450} sensor under the influence of interference gases extracted from Supplementary Figure 6c. Pulses #1-3 and #9-11 are 2% H_2, and pulses #4-8 are a mixture of 2% H_2 and **a** 5% CO_2 or **b** 5% CH_4 or **c** 0.2% CO. Source data are provided as a Source Data file.

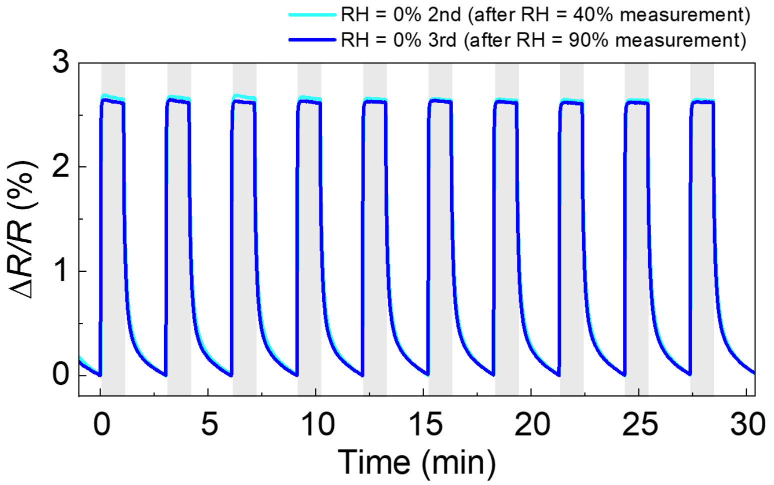

Supplementary Figure 32. Time-resolved *ΔR/R* response of the 20 nm C_{60}/5 nm PdCo/TAF/PMMA CHA_{450} to 10 pulses of 2% H_2 with relative humidities (RH) of 0%, measured after the RH test. Source data are provided as a Source Data file.

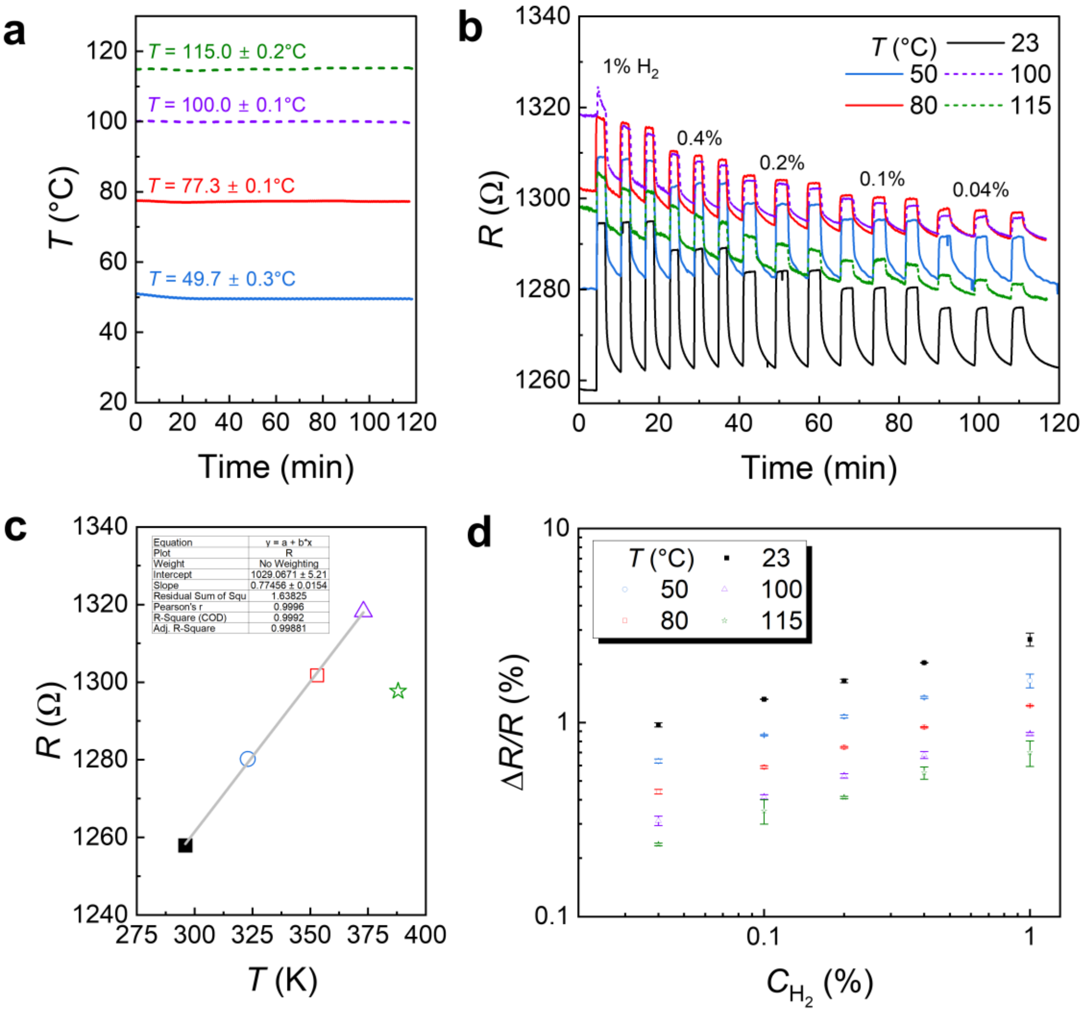

Supplementary Figure 33. **a** Measured temperatures at the sensor position during the tests. **b** Sensing responses of 20 nm C_{60}/5 nm PdCo CHA_{450} to stepwise decreasing H_2 concentrations with N_2 as the gas carrier and **c** extracted baseline resistance and **d** extracted sensitivity at different temperatures. The error bars in **d** indicate the standard deviation from 3 pulses. Data are presented as mean values +/- SD. Source data are provided as a Source Data file.

Supplementary Note 10. Scalability, feasibility, and potential challenges

The scalability, feasibility, and potential challenges related to the mass production and integration of our PdCo hexagonal nano-network sensors, as outlined below:

The fabrication process comprises four primary steps: (i) self-assembly of a polystyrene (PS) monolayer, (ii) reactive ion etching (RIE) to shape the nanosphere mask, (iii) physical vapor deposition (PVD) via glancing angle co-deposition (GLACD), and (iv) polymer coating via spin coating or thermal evaporation. These techniques are commonly used in micro/nanofabrication, and particularly RIE and PVD are compatible with standard semiconductor processing workflows. We believe our sensor design is inherently scalable and adaptable, making it a strong candidate for transitioning from laboratory-scale fabrication to industrial production. Nevertheless, we acknowledge the following potential challenges for real-world deployment:

(i) Uniformity over large areas: Achieving uniform PS monolayers and consistent polymer coatings across large areas is a key concern. Several techniques including spin coating, dip coating, and interfacial assembly have been employed to fabricate monolayers of PS nanospheres. Park et al. demonstrated the successful fabrication of a large-area PS monolayer (500 nm bead diameter, identical to our study) with nearly 95% coverage on a 5 cm × 5 cm glass substrate using spin coating and appropriate surfactants.[58] Lulek et al. reported large-area PS monolayer coating of 4- and 6-inch Si wafers using the air–water interface technique.[59] These self-assembly techniques are well-established and can be automated for scalable production. In addition, scalable coating methods such as slot-die coating, inkjet printing, and blade coating offer viable alternatives to spin coating for large-area fabrication.[60] Another approach for large scale production is implementing nanoimprint or UV lithography methods to achieve similar nanopatterns.

(ii) Material cost: Although Pd is an expensive material, the use of ultra-thin (~several nanometers) PdCo films reduces the total material cost to below 1 USD per sensor.

(iii) Sensor integration: Integration into packaged sensor systems (e.g., with electrical contacts, microheaters, wireless modules, etc.) requires compatibility with existing CMOS or MEMS technologies. In our case, the sensors are compact (1.0 cm × 0.5 cm) and lightweight, making them well-suited for integration into any commercial packaging. Furthermore, temperature control can be achieved locally using thermoelectric coolers (TECs) to mitigate environmental fluctuations and ensure consistent sensor performance.

(iv) Long-term stability and robustness: Maintaining structural and functional stability under real-world environmental conditions, such as variations in humidity, temperature cycling, and exposure to contaminants, is an important consideration. We are currently conducting ongoing research on various polymer encapsulation strategies,[61] local heating,[25] and thermal refreshing technique[51] to enhance sensor longevity under such conditions.

This combination of high performance, low material cost, and scalable fabrication makes our hydrogen sensor highly promising for future commercialization.

Supplementary references

1 Energy Efficiency and Renewable Energy (EERE), F. C. T. O. *Multi-Year Research, Development, and Demonstration Plan, 2011−2020. Section 3.7 Hydrogen Safety, Codes and Standards;*, <https://www.energy.gov/sites/prod/files/2015/06/f23/fcto_myrdd_safety_codes.pdf> (2015).

2 Energy Efficiency and Renewable Energy (EERE), E. E. a. R. E. *Funding Opportunity in Support of the Hydrogen Shot and a University Research Consortium on Grid Resilience*, <https://eere-exchange.energy.gov/> (2022).

3 Mandal, S. *et al.* A robust organic hydrogen sensor for distributed monitoring applications. *Nature Electronics* (2025). https://doi.org:10.1038/s41928-025-01352-y

4 Askar, P. *et al.* 1 ppm-detectable hydrogen gas sensor based on nanostructured polyaniline. *Scientific Reports* **14** (2024). https://doi.org:10.1038/s41598-024-77083-5

5 Mo, T. *et al.* High Response and ppb-Level Detection toward Hydrogen Sensing by Palladium-Doped α-Fe2O3 Nanotubes. *ACS Sensors* **9**, 5976-5984 (2024). https://doi.org:10.1021/acssensors.4c01829

6 Luong, H. M. *et al.* Ultra-fast and sensitive magneto-optical hydrogen sensors using a magnetic nano-cap array. *Nano Energy* **109**, 108332 (2023). https://doi.org:https://doi.org/10.1016/j.nanoen.2023.108332

7 Wen, L. *et al.* On-chip ultrasensitive and rapid hydrogen sensing based on plasmon-induced hot electron–molecule interaction. *Light: Science & Applications* **12**, 76 (2023). https://doi.org:10.1038/s41377-023-01123-4

8 Zhang, H. *et al.* 1ppm-detectable hydrogen gas sensors by using highly sensitive P+/N+ single-crystalline silicon thermopiles. *Microsystems & Nanoengineering* **9** (2023). https://doi.org:10.1038/s41378-023-00506-2

9 Zhang, X. *et al.* Ultralow detection limit and ultrafast response/recovery of the H2 gas sensor based on Pd-doped rGO/ZnO-SnO2 from hydrothermal synthesis. *Microsystems & Nanoengineering* **8** (2022). https://doi.org:10.1038/s41378-022-00398-8

10 Nugroho, F. A. A. *et al.* Inverse designed plasmonic metasurface with parts per billion optical hydrogen detection. *Nature Communications* **13** (2022). https://doi.org:10.1038/s41467-022-33466-8

11 Luong, H. M. *et al.* Sub-second and ppm-level optical sensing of hydrogen using templated control of nano-hydride geometry and composition. *Nature Communications* **12**, 2414-2414 (2021). https://doi.org:10.1038/s41467-021-22697-w

12 Tian, J. *et al.* A Ppb-level hydrogen sensor based on activated Pd nanoparticles loaded on oxidized nickel foam. *Sensors and Actuators B: Chemical* **329**, 129194 (2021). https://doi.org:https://doi.org/10.1016/j.snb.2020.129194

13 Pham, M. T. *et al.* Pd80Co20 Nanohole Arrays Coated with Poly(methyl methacrylate) for High-Speed Hydrogen Sensing with a Part-per-Billion Detection Limit. *ACS Applied Nano Materials* **4**, 3664-3674 (2021). https://doi.org:10.1021/acsanm.1c00169

14 Zhou, S. *et al.* Sub-10 parts per billion detection of hydrogen with floating gate transistors built on semiconducting carbon nanotube film. *Carbon* **180**, 41-47 (2021). https://doi.org:https://doi.org/10.1016/j.carbon.2021.04.076

15 Nugroho, F. A. A. *et al.* Metal–polymer hybrid nanomaterials for plasmonic ultrafast hydrogen detection. *Nature Materials* **18**, 489-495 (2019). https://doi.org:10.1038/s41563-019-0325-4

16 Darabpour, M. & Doroodmand, M. M. Fabrication of a Glow Discharge Plasma-Based Ionization Gas Sensor Using Multiwalled Carbon Nanotubes for Specific Detection of Hydrogen at Parts Per Billion Levels. *IEEE Sensors Journal* **15**, 2391-2398 (2015). https://doi.org:10.1109/JSEN.2014.2369738
17 Lee, J. S., Kim, S. G., Cho, S. & Jang, J. Porous palladium coated conducting polymer nanoparticles for ultrasensitive hydrogen sensors. *Nanoscale* **7**, 20665-20673 (2015). https://doi.org:10.1039/C5NR06193H
18 Shin, D. H. *et al.* Flower-like Palladium Nanoclusters Decorated Graphene Electrodes for Ultrasensitive and Flexible Hydrogen Gas Sensing. *Scientific Reports* **5**, 12294 (2015). https://doi.org:10.1038/srep12294
19 Moon, J., Hedman, H.-P., Kemell, M., Tuominen, A. & Punkkinen, R. Hydrogen sensor of Pd-decorated tubular TiO2 layer prepared by anodization with patterned electrodes on SiO2/Si substrate. *Sensors and Actuators B: Chemical* **222**, 190-197 (2016). https://doi.org:https://doi.org/10.1016/j.snb.2015.08.054
20 Luong, H. M. *et al.* Bilayer plasmonic nano-lattices for tunable hydrogen sensing platform. *Nano Energy* **71**, 104558 (2020). https://doi.org:https://doi.org/10.1016/j.nanoen.2020.104558
21 He, J. *et al.* Integrating plasmonic nanostructures with natural photonic architectures in Pd-modified <i>Morpho</i> butterfly wings for sensitive hydrogen gas sensing. *RSC Advances* **8**, 32395-32400 (2018). https://doi.org:10.1039/c8ra05046e
22 Nugroho, F. A. A., Darmadi, I., Zhdanov, V. P. & Langhammer, C. Universal Scaling and Design Rules of Hydrogen-Induced Optical Properties in Pd and Pd-Alloy Nanoparticles. *ACS Nano* **12**, 9903-9912 (2018). https://doi.org:10.1021/acsnano.8b02835
23 Luong, H. M. *et al.* Sub-second and ppm-level optical sensing of hydrogen using templated control of nano-hydride geometry and composition. *Nature Communications* **12**, 2414 (2021). https://doi.org:10.1038/s41467-021-22697-w
24 Lupan, O. *et al.* Ultra-sensitive and selective hydrogen nanosensor with fast response at room temperature based on a single Pd/ZnO nanowire. *Sensors and Actuators B: Chemical* **254**, 1259-1270 (2018). https://doi.org:https://doi.org/10.1016/j.snb.2017.07.200
25 Jo, M.-S. *et al.* Ultrafast (~0.6 s), Robust, and Highly Linear Hydrogen Detection up to 10% Using Fully Suspended Pure Pd Nanowire. *ACS Nano* **17**, 23649-23658 (2023). https://doi.org:10.1021/acsnano.3c06806
26 Bi, S. *et al.* High-performance palladium nanotube network as fast, high-resolution, and wide range hydrogen detector in atmosphere. *Sensors and Actuators B: Chemical* **404**, 135307 (2024). https://doi.org:https://doi.org/10.1016/j.snb.2024.135307
27 Lee, E. *et al.* Hydrogen gas sensing performance of Pd–Ni alloy thin films. *Thin Solid Films* **519**, 880-884 (2010). https://doi.org:https://doi.org/10.1016/j.tsf.2010.07.122
28 Koo, W.-T. *et al.* Accelerating Palladium Nanowire H2 Sensors Using Engineered Nanofiltration. *ACS Nano* **11**, 9276-9285 (2017). https://doi.org:10.1021/acsnano.7b04529
29 Cho, S.-Y. *et al.* Ultrasmall Grained Pd Nanopattern H2 Sensor. *ACS Sensors* **3**, 1876-1883 (2018). https://doi.org:10.1021/acssensors.8b00834
30 Hassan, K. & Chung, G.-S. Fast and reversible hydrogen sensing properties of Pd-capped Mg ultra-thin films modified by hydrophobic alumina substrates. *Sensors and Actuators*

B: Chemical **242**, 450-460 (2017).
https://doi.org:https://doi.org/10.1016/j.snb.2016.11.078
31 Rajoua, K., Baklouti, L. & Favier, F. Electronic and Mechanical Antagonist Effects in Resistive Hydrogen Sensors Based on Pd@Au Core–Shell Nanoparticle Assemblies Prepared by Langmuir–Blodgett. *The Journal of Physical Chemistry C* **119**, 10130-10139 (2015). https://doi.org:10.1021/acs.jpcc.5b01636
32 Zeng, X. Q. *et al.* Hydrogen Gas Sensing with Networks of Ultrasmall Palladium Nanowires Formed on Filtration Membranes. *Nano Letters* **11**, 262-268 (2011). https://doi.org:10.1021/nl103682s
33 Gao, M., Cho, M., Han, H.-J., Jung, Y. S. & Park, I. Palladium-Decorated Silicon Nanomesh Fabricated by Nanosphere Lithography for High Performance, Room Temperature Hydrogen Sensing. *Small* **14**, 1703691 (2018). https://doi.org:10.1002/smll.201703691
34 Shim, Y.-S. *et al.* Nanogap-controlled Pd coating for hydrogen sensitive switches and hydrogen sensors. *Sensors and Actuators B: Chemical* **255**, 1841-1848 (2018). https://doi.org:https://doi.org/10.1016/j.snb.2017.08.198
35 Chen, W. P. *et al.* Extraordinary room-temperature hydrogen sensing capabilities of porous bulk Pt–TiO2 nanocomposite ceramics. *International Journal of Hydrogen Energy* **41**, 3307-3312 (2016). https://doi.org:https://doi.org/10.1016/j.ijhydene.2015.12.151
36 Chen, R., Ruan, X., Liu, W. & Stefanini, C. A reliable and fast hydrogen gas leakage detector based on irreversible cracking of decorated palladium nanolayer upon aligned polymer fibers. *International Journal of Hydrogen Energy* **40**, 746-751 (2015). https://doi.org:https://doi.org/10.1016/j.ijhydene.2014.11.026
37 Lim, S. H. *et al.* Flexible Palladium-Based H2 Sensor with Fast Response and Low Leakage Detection by Nanoimprint Lithography. *ACS Applied Materials & Interfaces* **5**, 7274-7281 (2013). https://doi.org:10.1021/am401624r
38 Chung, M. G. *et al.* Flexible hydrogen sensors using graphene with palladium nanoparticle decoration. *Sensors and Actuators B: Chemical* **169**, 387-392 (2012). https://doi.org:https://doi.org/10.1016/j.snb.2012.05.031
39 Lee, J., Shim, W., Lee, E., Noh, J.-S. & Lee, W. Highly Mobile Palladium Thin Films on an Elastomeric Substrate: Nanogap-Based Hydrogen Gas Sensors. *Angewandte Chemie International Edition* **50**, 5301-5305 (2011). https://doi.org:10.1002/anie.201100054
40 Kiefer, T., Villanueva, L. G., Fargier, F., Favier, F. & Brugger, J. Fast and robust hydrogen sensors based on discontinuous palladium films on polyimide, fabricated on a wafer scale. *Nanotechnology* **21**, 505501 (2010). https://doi.org:10.1088/0957-4484/21/50/505501
41 Villanueva, L. G. *et al.* Highly ordered palladium nanodot patterns for full concentration range hydrogen sensing. *Nanoscale* **4**, 1964-1967 (2012). https://doi.org:10.1039/C2NR11983H
42 Behzadi pour, G. & Fekri aval, L. Highly sensitive work function hydrogen gas sensor based on PdNPs/SiO2/Si structure at room temperature. *Results in Physics* **7**, 1993-1999 (2017). https://doi.org:https://doi.org/10.1016/j.rinp.2017.06.026
43 Han, M., Jung, D. & Lee, G. S. Palladium-nanoparticle-coated carbon nanotube gas sensor. *Chemical Physics Letters* **610-611**, 261-266 (2014). https://doi.org:https://doi.org/10.1016/j.cplett.2014.07.053

44 Yun, S. & Ted Oyama, S. Correlations in palladium membranes for hydrogen separation: A review. *Journal of Membrane Science* **375**, 28-45 (2011). https://doi.org:https://doi.org/10.1016/j.memsci.2011.03.057
45 Wadell, C. *et al.* Hysteresis-Free Nanoplasmonic Pd–Au Alloy Hydrogen Sensors. *Nano Letters* **15**, 3563-3570 (2015). https://doi.org:10.1021/acs.nanolett.5b01053
46 Faiman, D. *et al.* Structure and optical properties of C60 thin films. *Thin Solid Films* **295**, 283-286 (1997). https://doi.org:https://doi.org/10.1016/S0040-6090(96)09043-8
47 Nguyen, T. D., Wang, F., Li, X.-G., Ehrenfreund, E. & Vardeny, Z. V. Spin diffusion in fullerene-based devices: Morphology effect. *Physical Review B* **87** (2013). https://doi.org:10.1103/physrevb.87.075205
48 Samad, B. A., Belanger, É. & Duguay, C. The effect of substrate deposition temperature on the electrical and optical properties of C60 thin films. *Journal of Applied Physics* **132** (2022). https://doi.org:10.1063/5.0099291
49 Morgan, C., Schmalbuch, K., García-Sánchez, F., Schneider, C. M. & Meyer, C. Structure and magnetization in CoPd thin films and nanocontacts. *Journal of Magnetism and Magnetic Materials* **325**, 112-116 (2013). https://doi.org:https://doi.org/10.1016/j.jmmm.2012.07.052
50 Östergren, I. *et al.* Highly Permeable Fluorinated Polymer Nanocomposites for Plasmonic Hydrogen Sensing. *ACS Applied Materials & Interfaces* **13**, 21724-21732 (2021). https://doi.org:10.1021/acsami.1c01968
51 Kim, K.-H. *et al.* Long-term reliable wireless H2 gas sensor via repeatable thermal refreshing of palladium nanowire. *Nature Communications* **15** (2024). https://doi.org:10.1038/s41467-024-53080-0
52 Anděra, V. & Bastl, Z. XPS and XAES study of the interaction of palladium and copper overlayers with fullerene films. *Czechoslovak Journal of Physics* **43**, 863-868 (1993). https://doi.org:10.1007/bf01595270
53 Ngene, P. *et al.* Polymer-Induced Surface Modifications of Pd-based Thin Films Leading to Improved Kinetics in Hydrogen Sensing and Energy Storage Applications. *Angewandte Chemie International Edition* **53**, 12081-12085 (2014). https://doi.org:10.1002/anie.201406911
54 Ai, B. *Glancing angle deposition*, <http://ailabcqu.com/GLAD?_l=en> (
55 Sartorius, S. *Surface area*, <https://www.mathworks.com/matlabcentral/fileexchange/62992-surface-area> (2024).
56 Wang, Y., Yang, F., Zhang, Z. & Zhao, Y. Performance of Transparent Metallic Thin Films. *The Journal of Physical Chemistry C* **125**, 16334-16342 (2021). https://doi.org:10.1021/acs.jpcc.1c04832
57 Lacy, F. Developing a theoretical relationship between electrical resistivity, temperature, and film thickness for conductors. *Nanoscale Research Letters* **6**, 636 (2011). https://doi.org:10.1186/1556-276x-6-636
58 Park, B., Na, S. Y. & Bae, I.-G. Uniform two-dimensional crystals of polystyrene nanospheres fabricated by a surfactant-assisted spin-coating method with polyoxyethylene tridecyl ether. *Scientific Reports* **9** (2019). https://doi.org:10.1038/s41598-019-47990-z
59 Lulek, E. & Ertas, Y. N. Simple and Rapid Monolayer Self-Assembly of Nanoparticles at the Air/Water Interface. *Langmuir* **40**, 18039-18048 (2024). https://doi.org:10.1021/acs.langmuir.4c01622

60 Chen, C. *et al.* Screen-Printing Technology for Scale Manufacturing of Perovskite Solar Cells. *Advanced Science* **10** (2023). https://doi.org:10.1002/advs.202303992
61 Darmadi, I., Nugroho, F. A. A. & Langhammer, C. High-Performance Nanostructured Palladium-Based Hydrogen Sensors—Current Limitations and Strategies for Their Mitigation. *ACS Sensors* **5**, 3306-3327 (2020). https://doi.org:10.1021/acssensors.0c02019
