# Supplementary material for: Fullerene-decorated PdCo nano-resistor network hydrogen sensors with sub-second response and parts-per-billion detection at room temperature
Source: Nat Commun. 2025 Dec 19;17:977. doi: 10.1038/s41467-025-67708-2 (PMC12847907; doi:10.1038/s41467-025-67708-2)
Supplement: Supplementary file 3 — Reporting Summary [file 41467_2025_67708_MOESM3_ESM.pdf]

nature portfolio

Corresponding author(s): Tu Anh Ngo, Tho D. Nguyen

Last updated by author(s): 2025/10/27

Reporting Summary

Nature Portfolio wishes to improve the reproducibility of the work that we publish. This form provides structure for consistency and transparency in reporting. For further information on Nature Portfolio policies, see our Editorial Policies and the Editorial Policy Checklist.

Statistics

For all statistical analyses, confirm that the following items are present in the figure legend, table legend, main text, or Methods section.

n/a	Confirmed	
☐	☒	The exact sample size (*n*) for each experimental group/condition, given as a discrete number and unit of measurement
☐	☒	A statement on whether measurements were taken from distinct samples or whether the same sample was measured repeatedly
☐	☒	The statistical test(s) used AND whether they are one- or two-sided<br>*Only common tests should be described solely by name; describe more complex techniques in the Methods section.*
☐	☒	A description of all covariates tested
☐	☒	A description of any assumptions or corrections, such as tests of normality and adjustment for multiple comparisons
☐	☒	A full description of the statistical parameters including central tendency (e.g. means) or other basic estimates (e.g. regression coefficient) AND variation (e.g. standard deviation) or associated estimates of uncertainty (e.g. confidence intervals)
☒	☐	For null hypothesis testing, the test statistic (e.g. *F*, *t*, *r*) with confidence intervals, effect sizes, degrees of freedom and *P* value noted<br>*Give P values as exact values whenever suitable.*
☒	☐	For Bayesian analysis, information on the choice of priors and Markov chain Monte Carlo settings
☒	☐	For hierarchical and complex designs, identification of the appropriate level for tests and full reporting of outcomes
☒	☐	Estimates of effect sizes (e.g. Cohen's *d*, Pearson's *r*), indicating how they were calculated

Our web collection on statistics for biologists contains articles on many of the points above.

Software and code

Policy information about availability of computer code

Data collection	LabVIEW 2012 (Version 12.0.1f5)
Data analysis	OriginPro 2024b 10.1.5.132 (Student Version), MATLAB R2021b Update 6, Gwyddion 2.61, Park Sytems XEI 4.3.4.Build22.

For manuscripts utilizing custom algorithms or software that are central to the research but not yet described in published literature, software must be made available to editors and reviewers. We strongly encourage code deposition in a community repository (e.g. GitHub). See the Nature Portfolio guidelines for submitting code & software for further information.

Data

Policy information about availability of data

All manuscripts must include a data availability statement. This statement should provide the following information, where applicable:

- Accession codes, unique identifiers, or web links for publicly available datasets
- A description of any restrictions on data availability
- For clinical datasets or third party data, please ensure that the statement adheres to our policy

Source data are provided with this paper. Additional information can be obtained from the corresponding authors upon request.

Research involving human participants, their data, or biological material

Policy information about studies with human participants or human data. See also policy information about sex, gender (identity/presentation), and sexual orientation and race, ethnicity and racism.

Reporting on sex and gender	n/a
Reporting on race, ethnicity, or other socially relevant groupings	n/a
Population characteristics	n/a
Recruitment	n/a
Ethics oversight	n/a

Note that full information on the approval of the study protocol must also be provided in the manuscript.

Field-specific reporting

Please select the one below that is the best fit for your research. If you are not sure, read the appropriate sections before making your selection.

☒ Life sciences ☐ Behavioural & social sciences ☐ Ecological, evolutionary & environmental sciences

For a reference copy of the document with all sections, see nature.com/documents/nr-reporting-summary-flat.pdf

Life sciences study design

All studies must disclose on these points even when the disclosure is negative.

Sample size	For structural calibration experiments (e.g., PdCo and C60 thickness dependence, PdCo stoichiometry, and nanohole size dependence), we used at least three to six independent variations (N = 3–6) for each parameter to capture systematic trends in hydrogen sensing performance. After identifying the optimal configuration, we fabricated a minimum of three independent sensors (N ≥ 3) with the selected design to confirm reproducibility of sensitivity, response/recovery times, and baseline stability. This approach ensured that both parameter-dependent trends and final device performance were robust. No formal power calculation was performed, as the goal was to demonstrate reproducibility and trends rather than population-level hypothesis testing.
Data exclusions	No data were excluded from the analysis except for clearly defective devices (e.g., discontinuous nano-network). These exclusion criteria were pre-established before measurements and applied uniformly across all experiments.
Replication	All key findings including sensitivity, response/recovery time versus hydrogen partial pressure were replicated with at least three independently fabricated sensors per device type. All replicates showed consistent trends within experimental uncertainty, confirming reproducibility of the results.
Randomization	Sensors with different PdCo stoichiometry, PdCo and C60 thickness, and nanohole size were fabricated in separate groups, but their testing order was randomly assigned. Randomizing the order of measurements helped minimize potential bias from time-dependent factors such as chamber base pressure drift, sensor aging, or temperature fluctuations. For hysteresis test, the sequence of hydrogen partial pressures was alternated between increasing and decreasing order across replicates to mitigate history effects.
Blinding	Blinding was not relevant to this study because the sensing response (electrical resistance change) was recorded automatically by the data acquisition system and analyzed using pre-defined scripts, eliminating operator bias in data collection or analysis.

Reporting for specific materials, systems and methods

We require information from authors about some types of materials, experimental systems and methods used in many studies. Here, indicate whether each material, system or method listed is relevant to your study. If you are not sure if a list item applies to your research, read the appropriate section before selecting a response.

Materials & experimental systems

n/a	Involved in the study
☒	☐ Antibodies
☒	☐ Eukaryotic cell lines
☒	☐ Palaeontology and archaeology
☒	☐ Animals and other organisms
☒	☐ Clinical data
☒	☐ Dual use research of concern
☒	☐ Plants

Methods

n/a	Involved in the study
☒	☐ ChIP-seq
☒	☐ Flow cytometry
☒	☐ MRI-based neuroimaging

Plants

Seed stocks	n/a
Novel plant genotypes	n/a
Authentication	n/a
